# Synergistic therapeutic effects of intracerebral transplantation of human modified bone marrow-derived stromal cells (SB623) and voluntary exercise with running wheel in a rat model of ischemic stroke

**DOI:** 10.1186/s13287-023-03236-4

**Published:** 2023-01-24

**Authors:** Satoru Yabuno, Takao Yasuhara, Takayuki Nagase, Satoshi Kawauchi, Chiaki Sugahara, Yosuke Okazaki, Kakeru Hosomoto, Susumu Sasada, Tatsuya Sasaki, Naoki Tajiri, Cesar V. Borlongan, Isao Date

**Affiliations:** 1 Department of Neurological Surgery, Faculty of Medicine, Dentistry and Pharmaceutical Sciences, Okayama University, 2-5-1 Shikata-Cho, Kita-Ku, Okayama, 700-8558 Japan; 2 Department of Neurophysiology and Brain Science, Nagoya City University Graduate School of Medical Sciences and Medical School, Nagoya, Japan; 3 Department of Neurosurgery and Brain Repair, Morsani College of Medicine, University of South Florida, Tampa, FL USA

**Keywords:** Cerebral ischemic infarct, Rehabilitation, Regenerative medicine, SB623, Voluntary exercise

## Abstract

**Background:**

Mesenchymal stromal cell (MSC) transplantation therapy is a promising therapy for stroke patients. In parallel, rehabilitation with physical exercise could ameliorate stroke-induced neurological impairment. In this study, we aimed to clarify whether combination therapy of intracerebral transplantation of human modified bone marrow-derived MSCs, SB623 cells, and voluntary exercise with running wheel (RW) could exert synergistic therapeutic effects on a rat model of ischemic stroke.

**Methods:**

Wistar rats received right transient middle cerebral artery occlusion (MCAO). Voluntary exercise (Ex) groups were trained in a cage with RW from day 7 before MCAO. SB623 cells (4.0 × 10^5^ cells/5 μl) were stereotactically injected into the right striatum at day 1 after MCAO. Behavioral tests were performed at day 1, 7, and 14 after MCAO using the modified Neurological Severity Score (mNSS) and cylinder test. Rats were euthanized at day 15 after MCAO for mRNA level evaluation of ischemic infarct area, endogenous neurogenesis, angiogenesis, and expression of brain-derived neurotrophic factor (BDNF) and vascular endothelial growth factor (VEGF). The rats were randomly assigned to one of the four groups: vehicle, Ex, SB623, and SB623 + Ex groups.

**Results:**

SB623 + Ex group achieved significant neurological recovery in mNSS compared to the vehicle group (*p* < 0.05). The cerebral infarct area of SB623 + Ex group was significantly decreased compared to those in all other groups (*p* < 0.05). The number of BrdU/Doublecortin (Dcx) double-positive cells in the subventricular zone (SVZ) and the dentate gyrus (DG), the laminin-positive area in the ischemic boundary zone (IBZ), and the mRNA level of BDNF and VEGF in SB623 + Ex group were significantly increased compared to those in all other groups (*p* < 0.05).

**Conclusions:**

This study suggests that combination therapy of intracerebral transplantation SB623 cells and voluntary exercise with RW achieves robust neurological recovery and synergistically promotes endogenous neurogenesis and angiogenesis after cerebral ischemia, possibly through a mechanism involving the up-regulation of BDNF and VEGF.

**Supplementary Information:**

The online version contains supplementary material available at 10.1186/s13287-023-03236-4.

## Background

Stroke is the leading global cause of long-term disability and death, with 25.7 million survivors of stroke [1]. About 70% of these strokes are of ischemic type and remain the most common form of stroke [2]. Although the treatment of acute ischemic stroke has been developed such as drug thrombolysis and mechanical thrombectomy [3, 4], approximately 90% of ischemic stroke patients have not received these treatments largely due to their narrow therapeutic window [5]. Therefore, developing safe and effective therapies beyond the acute phase is warranted to clinically impact stroke treatment.

Cell transplantation therapy has emerged as a potential candidate treatment for ischemic stroke. In particular, adult bone marrow-derived mesenchymal stromal cells (MSCs) are attractive because of their relative ease in procurement from voluntary adult donors and potential for a multi-pronged action involving the regeneration of neural networks by stimulating the replacement of damaged cells, secretion of neurotrophic factors, and enhanced neurogenesis in experimental stroke studies [6–9].

SB623 cells are human modified bone marrow-derived MSCs transiently transfected with the human *Notch-1* intracellular domain. Transplantation of SB623 cells has been shown to reduce infarct area and improve performance in behavioral tests in rats with experimental ischemic stroke [10]. Several studies have demonstrated that treatment with SB623 cells up-regulates neurotrophic factors [11], promotes angiogenesis [12], and enhances the migration and differentiation of neural stem cells into the damaged area [13]. Furthermore, in clinical trials intracerebral transplantation of SB623 cells was safe for patients suffering from chronic ischemic stroke (STR-01) [14, 15] and traumatic brain injury (STEMTRA trial): in the latter indication there was also a significant improvement in motor function [16].

Rehabilitation therapy is one of the standard treatments for stroke patients [17]. In studies in animal models of stroke, voluntary exercise with running wheel (RW) and enriched environment (EE) promoted functional recovery by enhancing neurogenesis characterized by increased endogenous cell proliferation in the subventricular zone (SVZ) [18–20].

Based on the converging target of neurogenesis by stem cell transplantation and physical rehabilitation therapy, it is tempting to explore the combination of both treatments to potentially further enhance functional outcomes in stroke. Indeed, the combination of cell transplantation and voluntary or forced exercise was investigated in animal models of stroke and cerebral palsy [21–28]. However, the specific effects of SB623 as a donor for cell transplantation in combination with voluntary exercise with RW in a rat model of ischemic stroke have yet to be demonstrated. The present study aimed to examine whether this combination therapy could synergistically achieve functional recovery and promote endogenous neurogenesis and angiogenesis, and whether these effects could be mediated by neurotrophic factors in a rat model of ischemic stroke. This study was conducted in line with recent Stem Cell Therapies as an Emerging Paradigm in Stroke (STEPS) recommendations [29, 30]. The results of this translational research showed a benefit of combination therapy using SB623 cell intracerebral transplantation and rehabilitation in ischemic stroke patients.

## Materials and methods

### Ethics statement

This study was conducted in accordance with the guidelines of the Institutional Animal Care and Use Committee of Okayama University Faculty of Medicine. The protocol was specifically approved by the Institutional Animal Care and Use Committee of Okayama University Faculty of Medicine (protocol #OKU-202036). All rats were euthanized with an overdose of a mixed solution of 0.3 mg/kg of medetomidine, 4.0 mg/kg of midazolam, and 5.0 mg/kg of butorphanol delivered via an intraperitoneal injection at the end of the protocols described below. For MCAO surgery, anesthesia was induced by inhalation of 2.0% sevoflurane in 30% O_2_ and 70% N_2_O through a facial mask. All efforts were made to minimize animals’ distress. Measurements and analyses were performed by examiners blinded to the treatment groups of rats in this study.

### Animals

Adult male Wistar rats (Jackson Laboratory Japan, Inc., Yokohama, Japan) weighing 280 to 320 g (8 weeks old) at the beginning of the experiment were used as subjects in this study. The animal housing facility was maintained on 12 h light/dark cycle, and the animals had free access to food and water. Body weight was recorded at day − 7, 0, 1, 7, and 14 after transient middle cerebral artery occlusion (MCAO). Immunosuppressant was not used in this study because of the immunosuppressive potency of SB623 cells [10, 11, 31].

### Rats and experimental design

**Fig. 1:**
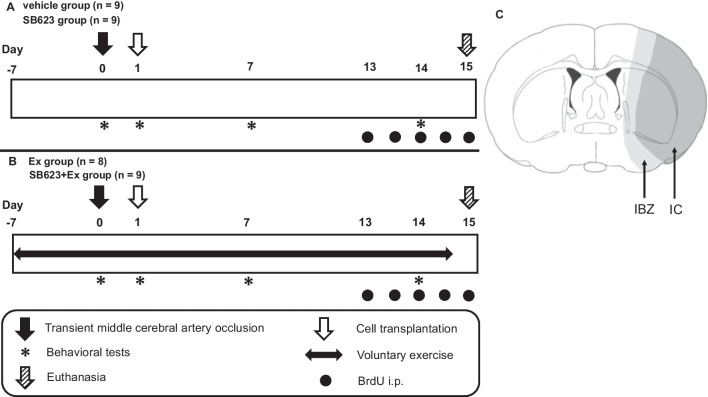
Experimental protocol of this study and illustration of ischemic lesion. **a** Study design for vehicle group (*n* = 9) and SB623 group (*n* = 9). **b** Study design for Ex group (*n* = 8) and SB623 + Ex group (*n* = 9). The Ex group and the SB623 + Ex group trained in a cage with RW from 7 days before MCAO and continued for 21 days. All rats were performed right MCAO. At day 1 after MCAO, all rats which were diagnosed with ischemic stroke by behavioral test were stereotactically transplanted with DMEM or SB623 cells into the right striatum. At day 15 after MCAO, all rats were euthanized, followed by histological analysis and assessment of the mRNA expression of BDNF and VEGF in the IBZ via quantitative reverse transcription polymerase chain reaction analysis. **c** A coronal section identified at the level of 0.3 mm posterior from the bregma in rat brain that divides the right ischemic hemisphere into two subregions (ischemic core [IC]; ischemic boundary zone [IBZ]). Abbreviation: RW: running wheel; MCAO: middle cerebral artery occlusion; DMEM: Dulbecco’s modified Eagle’s medium; BDNF: brain-derived neurotrophic factor; VEGF: vascular endothelial growth factor, IBZ: ischemic boundary zone

All rats received MCAO and were randomly assigned to one of the four groups: Dulbecco’s modified Eagle’s medium (DMEM [Sigma-Aldrich, St. Louis, Missouri, USA]) injection group (vehicle group: *n* = 9), DMEM injection + voluntary exercise group (Ex group: *n* = 8), SB623 cell transplantation group (SB623 group: *n* = 9), and combination therapy of SB623 cell transplantation + voluntary exercise group (SB623 + Ex group: *n* = 9) (see study design in Fig. 1).

**Fig. 2:**
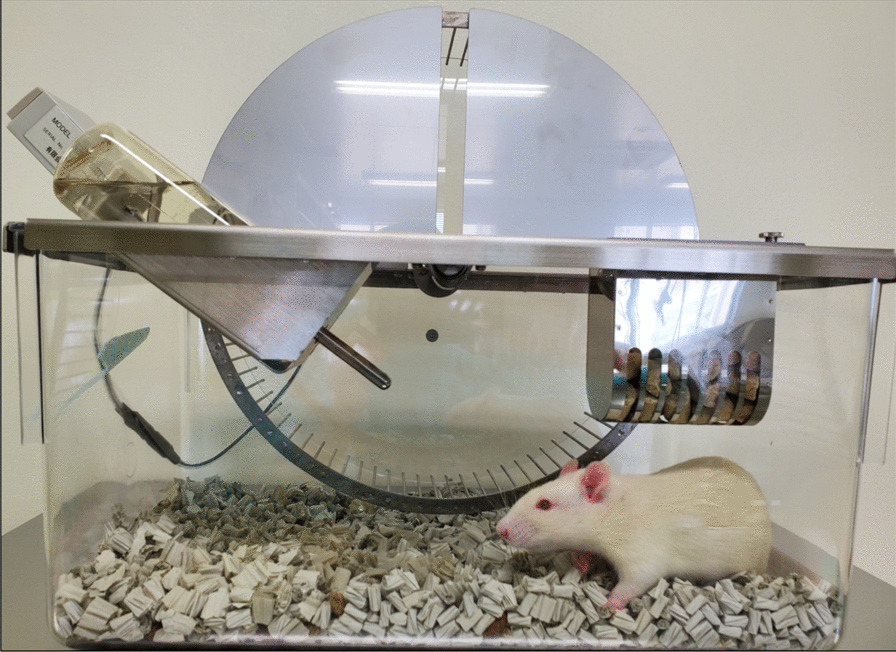
Running wheel used in this study. The Ex group and the SB623 + Ex group were individually housed in cages with RW for 7 days before MCAO and freely exercised until day 14 after MCAO. *Abbreviation*: RW: running wheel; MCAO: middle cerebral artery occlusion

At day 7 before MCAO, rats in the vehicle and SB623 groups were housed individually in standard cages, while rats in the Ex and SB623 + Ex groups were housed in a cage with a RW (Fig. 2). Accordingly, the voluntary RW exercise started in rats in the Ex and SB623 + Ex groups at 7 days before MCAO and continued for 21 days. Only one rat was excluded from the Ex group, because the rat was euthanized and excluded due to uncontrolled bleeding after MCAO surgery.

### Running wheel apparatus

The voluntary exercise groups were individually maintained in plastic cages containing animal paper bedding and running wheels (SWY-30, Melquest, Toyama, Japan, 310 Φ × 84 mm, perimeter = 1 m). The number of rotations of RW was measured by a porcelain sensor and converted to distance (meters). The distance was shown on the attached display counter (CNT-10, Melquest, Toyama, Japan) and we recorded the distance for 24 h from 10:00 am every day at day − 7 to day 14 after MCAO. The rats had free access to RW as well as food and water.

### SB623 cell production

Human modified bone marrow-derived MSCs, called SB623 cells, were produced by transient transfection of a plasmid encoding the human *Notch-1* intracellular domain cDNA to bone marrow-derived MSCs obtained from a healthy young adult human donor (SanBio, Inc., Mountain View, CA, USA). SB623 cells were characterized by previous studies [11, 29, 32, 33]. Yasuhara et al. and Tate et al. applied these cells to animal models of ischemic stroke and Parkinson’s disease, respectively [10, 34].

### SB623 cell preparation

Frozen vials containing SB623 cells provided by SanBio, Inc., were gently thawed at 37 °C water baths. Phosphate-buffered saline (PBS, 10 ml) was added to 1 ml of SB623 cell suspension and mixed by gently pipetting. The suspension was then centrifuged at 1000 rpm (200 × g) for 8 min at room temperature to form a pellet of cells. After removing the supernatant liquid without disturbing the pellet, they were dissolved in 100 μl of DMEM without serum and antibiotics. Viability was measured using the Trypan blue dye exclusion method and cell concentration was adjusted to 8.0 × 10^4^ cells/μl for transplantation. The viability range was 95.2–98.3% (average 96.6 ± 1.4%) before transplantation. Following completion of the transplantation procedure within 3 h post-thaw, the viability range of the remaining cells was found to be 83.6–98.9% (average 91.4 ± 6.5%). The concentration of SB623 cells used in our study (8.0 × 10^4^ cells/μl) was set close to that used in clinical trials (8.0 × 10^3^–3.3 × 10^4^ cells/μl) [14, 16] which resulted in a cell solution of 4.0 × 10^5^ cells/5 μl.

### Surgical procedures to induce transient middle cerebral artery occlusion

Transient middle cerebral artery occlusion (MCAO) was carried out according to the intraluminal suture method as in our previous studies [35]. Throughout all surgical procedures, body temperature was maintained at 37 °C with a heating pad. Under general anesthesia (2% sevoflurane in 70% N_2_O and 30% O_2_), the bifurcation of the right common carotid artery was exposed. After the right external carotid artery (ECA) was cut, a 4–0 monofilament nylon suture with a silicone-coated tip (Xantopren L blue and ACTIVATOR 2 Universal Liquid, Heraeus Kulzer GmbH & Co. KG, Hanau, Germany) was inserted for 18–20 mm into the lumen of internal carotid artery (ICA) and advanced toward the origin of the right MCA until mild resistance was felt. After 90 min of occlusion, the filament was withdrawn and the ECA was cauterized. At the end of the operation, the skin was closed with 3–0 silk sutures. To relieve pain and discomfort in the postoperative period, topical 1% lidocaine (10 mg/kg) was injected around the wound, and carprofen (5 mg/kg) was injected subcutaneously for postoperative analgesia immediately after surgery. During recovery from anesthesia, the animals were returned to their home cages.

### SB623 cells intracerebral transplantation

All rats were anesthetized with a mixed solution of 0.3 mg/kg of medetomidine, 4.0 mg/kg of midazolam, and 5.0 mg/kg of butorphanol that was intraperitoneally injected. The individual animals were fixed in a stereotactic instrument (Narishige, Japan). Transplantation of SB623 cells (8.0 × 10^4^ cells/μl) or DMEM solution targeted the right striatum using a 22-gauge Hamilton syringe. The injection coordinates were as follows: 1.0 mm anterior to the bregma, 3.0 mm lateral to the sagittal suture, and 5.0 mm ventral to the surface of the brain with the tooth bar set at 0.0 mm. SB623 cells were prepared as single-cell suspension at a density of 8.0 × 10^4^ cells/µl and a dose of 5 µl (4.0 × 10^5^ cells in total) were injected at a rate of 1 μl/min. After the injection, the syringe was left in place for an additional 2 min, followed by being retracted slowly (1 mm/min). The DMEM used for injection or cell formulation was not supplemented with serum and antibiotics.

### Behavioral tests

#### Modified neurological severity score (mNSS)

The mNSS was evaluated at day − 7, 0, 1, 7, and 14 after MCAO. This score assessed motor function, sensory disturbance, balance, and reflex tests. In this score, one score point was awarded for the inability to perform the test or for the lack of reflex. Therefore, a higher score indicates more severe injury. Neurological function was graded on a scale of 0 to 18 (normal score: 0; maximal deficit score: 18) [35–37]. To standardize the postsurgical neurological severity of the enrolled subjects, only rats scored 7–12 points of mNSS on the following day after MCAO were included in this study.

#### Cylinder test

To assess the degree of forepaw asymmetry, the cylinder test was performed at day 0, 1, 7, and 14 after MCAO. A baseline behavioral test was performed before MCAO at day 0 to obtain baseline data of the motor performance before ischemic stroke was induced. Rats were placed in a transparent cylinder (diameter 20 cm, height 30 cm) for 3 min and the number of forepaw contacts to the cylinder wall was counted [38–40]. The score of the cylinder test on this study reflected a contralateral bias, that is, ([number of contacts with contralateral limb]—[number of contacts with ipsilateral limb]/[number of total contacts] × 100) [35, 41]. The behavioral tests were always performed by the same two investigators blinded to treatments at the same place with no outside noises and low light.

### Histological analyses

#### Measurement of the cerebral infarct area

All rats were euthanized with an overdose of a mixed solution of 0.3 mg/kg of medetomidine, 4.0 mg/kg of midazolam, and 5.0 mg/kg of butorphanol at day 15 after MCAO, and perfused transcardially with 200 ml of cold PBS and 200 ml of 4% paraformaldehyde (PFA) in PBS. Brains were removed and post-fixed in the same fixative overnight at 4 °C, and subsequently stored on 30% sucrose in PBS until completely submerged. Coronal sections of 30 μm thickness were prepared with a freezing microtome ( − 20 °C). These sections were mounted onto slides. Five rats from each group were randomly selected. Nissl staining was performed to evaluate the cerebral infarct area. The cerebral infarct area was measured at the site of cell transplantation using a computerized image analysis using ImageJ (National Institutes of Health, Bethesda, USA). We evaluated cerebral infarct area ratio by the following method: Cerebral infarct area ratio = [LT – (RT – RI)] × 100/LT (%), where LT is the area of the left hemisphere in mm^2^, RT is the area of the right hemisphere in mm^2^, and RI is the infarct area in mm^2^ [35, 42, 43]. Figure 1c depicts a standard coronal section identified at the level of 0.3 mm posterior from the bregma in rat brain, which divides the right hemisphere into two subregions (ischemic core [IC] and ischemic boundary zone [IBZ]).

#### 2, 3, 4-triphenyltetrazolium chloride (TTC) staining

Another cohort of rats (*n* = 8) in each group were euthanized following saline perfusion and brains were quickly removed at 7 days after MCAO for TTC staining to illustrate the ischemic lesion. Thereafter, six consecutive coronal sections of 2 mm thickness were prepared. Brain slices were incubated in a 0.2% solution of TTC (Kanto Chemistry Co., Tokyo, Japan) in PBS at 37 °C for 30 min and immersion in 4% buffered formaldehyde solution. The normal area of brain was stained dark red based on intact mitochondrial function, whereas the infarct area remained unstained [42]. The stained brain sections were captured with a digital camera. The evaluation method of cerebral infarct area ratio was the same as Nissl staining.

#### 5-bromo2′-deoxyuridine (BrdU) labeling

In order to evaluate the endogenous neurogenesis in the subventricular zone (SVZ) and the dentate gyrus (DG) of hippocampus, 5-bromo2′-deoxyuridine (BrdU, NACALAI TESQUE INC., Kyoto, Japan) was injected into all rats at a concentration of 50 mg/kg body weight, with five consecutive intraperitoneal injections every 12 h from day 13 to 15 after MCAO before euthanasia to label proliferative cells [44, 45].

#### Immunofluorescent staining for BrdU, laminin, and STEM121

Five rats from each group were randomly selected. To demonstrate the endogenous neurogenesis and cell proliferation in the SVZ and the DG, BrdU/Doublecortin (Dcx)/4,6-diamidino-2-phenylindole (DAPI) triple-immunofluorescent staining was performed based on methods from previous reports [44, 46]. Briefly, free-floating sections were first incubated in HCl (2 N, 37 °C) for 20 min. This was followed by sodium borate incubation (pH 8.5) for 10 min. After rinsing four times with PBS, the sections were incubated for 24 h at 4 °C with rat anti-BrdU antibody (1:100, OBT0030G; Bio-Rad Laboratories Inc., Hercules, California, USA), rabbit anti-Dcx antibody (1:200, #4604; Cell Signaling Technology, Danvers, Massachusetts, USA), 10% normal horse serum (Invitrogen, Carlsbad, California, USA) and 0.1% TritonX (NACALAI TESQUE INC. Kyoto, Japan). After rinsing several times in PBS, the sections were incubated for 90 min with biotinylated anti-rat secondary antibody (1:100, 712–065-153; Jackson ImmunoResearch Laboratories Inc., West Grove, Pennsylvania, USA). Then, the sections were washed four times in PBS and incubated for 1 h with Streptavidin Alexa-488 (1:200, S11223; Invitrogen), goat anti-rabbit IgG Cy3 (1:200, ab97075; Abcam, Cambridge, UK), and DAPI (1:500, D3751; Thermo Fisher Scientific, Waltham, Massachusetts, USA). Finally, the sections were washed four times in PBS and mounted on albumin-coated glass slides.

Next, we performed the immunofluorescent staining for laminin and double-immunofluorescent staining for STEM121/DAPI to evaluate the formation of new blood vessels about the angiogenesis and the viability of SB623 cells. In order to reveal cell viability via STEM121 immunostaining, we transplanted SB623 cells into the right striatum of eight-week healthy and ischemic stroke male rats (healthy rat: *n* = 18; ischemic stroke rat: *n* = 6) with consequent euthanasia at day 3, 7, and 14 after transplantation, respectively (each group: healthy rat: *n* = 6; ischemic stroke rat: *n* = 2). SB623 cells were transplanted and rats underwent MCAO using the same procedures as described above.

We washed 30-μm-thick sections four times in PBS and incubated them in 10% normal horse serum and primary antibody: rabbit anti-laminin antibody (1:500; AB11575, Abcam, Cambridge, UK), mouse monoclonal antihuman STEM121 (1:500; Y40410, Takara Bio, Inc., Shiga, Japan) overnight at 4 °C. Next, we washed the sections four times in PBS, incubated them for 1 h in Fluorescein (FITC) AffiniPure Donkey Anti-Rabbit IgG (H + L) (1:200; 711-095-152, Jackson ImmunoResearch Laboratories Inc., West Grove, Pennsylvania, USA) and goat anti-mouse IgG H&L Alexa Fluor® 594 (1:500; ab150116, Abcam, Cambridge, UK), and DAPI (1:500) in a dark chamber, then washed them four times in PBS and finally mounted and embedded them in albumin-coated glass slides.

#### Cell counting

Quantification of BrdU/Dcx double-positive cells in the SVZ and the DG was performed using a method according to previous studies [44, 46]. Briefly, BrdU/Dcx double-positive cells were counted bilaterally in four defined areas (200 × 60 μm) of the lateral ventricle wall and eight defined areas (200 × 40 μm) of the subgranular cell zone (SGZ) of DG.

For cell counting, the sections used for analyses were taken from the brain at the level of SVZ in the area of 0.0–0.9 mm posterior from the bregma and DG in the area of 3.3–4.2 mm posterior from the bregma. The cell number was counted bilaterally and averaged on 5 sections (every 6th section). In summary, we counted 16 areas (4 areas × 2 sections × 2 hemispheres) for the SVZ (Additional file 1: Fig. S1a), and 32 areas (8 areas × 2 sections × 2 hemispheres) for the DG (Additional file 1: Fig. S1b) in each rat. Additionally, both Nissl staining and immunofluorescent staining were visualized using a BZ-X810 (Keyence, Osaka, Japan).

In addition, we measured the area of laminin-positive structures of randomly captured images (500 × 500 μm square) of ischemic cortex from two different ischemic boundary zone (IBZ) slices (from 0.0 to 0.5 mm anterior to the bregma) and the ratio to the non-ischemic side was evaluated (Additional file 1: Fig. S2). For the quantification, these areas were measured with ImageJ software (National Institutes of Health, Bethesda, USA) and used the averages for statistical analyses. To estimate the number of SB623 cells, STEM121/DAPI double-positive cells were counted in the SB623 cells transplanted area of every six sectioned slices and calculated for overall cell viability [10].

### Reverse transcription polymerase chain reaction and quantitative real-time PCR (qRT-PCR)

In order to explore the potential mechanism underlying the combination of SB623 cell transplantation and exercise with RW, we assessed the mRNA expression of brain-derived neurotrophic factor (BDNF) and vascular endothelial growth factor (VEGF) in the IBZ via quantitative reverse transcription polymerase chain reaction analysis. Another cohort of 24 rats were euthanized at day 15 after MCAO (each group: *n* = 6). The brains were immediately removed and the tissue corresponding to the IBZ was dissected for qRT-PCR. These tissues were not perfused with saline before dissecting the region to be analyzed. The RNA was extracted with RNeasy® Mini Kit (QIAGEN, SAS, France). Total RNA was isolated from the IBZ, and cDNA was synthesized with a High-Capacity cDNA Reverse Transcription Kit (Invitrogen, Carlsbad, CA, USA). The qRT-PCR was performed using the cDNA for RNA quantitation with SYBR Green PCR Master Mix and analyzed using the StepOnePlus ™ System (Life Technologies, Carlsbad, CA, USA). The primers for qRT-PCR were designed using the Primer3Plus software, and the primer sequences are listed in Table 1. The expression of GAPDH mRNA was used as an internal control. The 2^−ΔΔCT^ method was used to analyze the relative gene expression data [47].

**Table 1:** Primers used in real-time polymerase chain reaction

Gene	Sequence (5′-3′)	Product size (bp)
BDNF	Forward: TTC GGC CCA ACG AAG AAA AC	331
	Reverse: TAG ACA TGT TTG CGG CAT CC	
VEGF	Forward: GGT TGC TCC TTC ACT CCC TC	141
	Reverse: TTC TCC CCT CTC TTC TCG GG	
GAPDH	Forward: ATG CCC CCA TGT TTG TGA TG	136
	Reverse: TCC ACG ATG CCA AAG TTG TC	

*BDNF*: brain-derived neurotrophic factor, *VEGF*: vascular endothelial growth factor, *GAPDH*: glyceraldelyde-3-phosphate dehydrogenase

### Statistical analyses

We used the IBM SPSS 20.0 (SPSS, Chicago, IL, USA). The results of running distance were analyzed by a two-tailed* t* test. Comparisons between running distance and changes over time were analyzed using repeated measures analysis of variance (ANOVA) with the post hoc Bonferroni correction test. Additionally, the results of body weight, behavioral tests, immunofluorescent staining, and qRT-PCR were analyzed using a one-way ANOVA with a Tukey’s post hoc test to compare differences between groups (after normal distribution of the data had been confirmed using a Shapiro–Wilk normality test). To investigate the relationship between the results of the immunofluorescent staining for the endogenous neurogenesis in SVZ and DG, the cerebral infarct area ratio, and the running distance, the Pearson’s product–moment correlation coefficient was calculated. All quantitative results except running distance were presented as mean ± standard error (SE). The results of running distance were presented as mean ± standard deviation (SD). Statistical significance was present at* p* value < 0.05.

## Results

### Voluntary exercise reduces body weight at day 0 and day 1 after MCAO

**Fig. 3:**
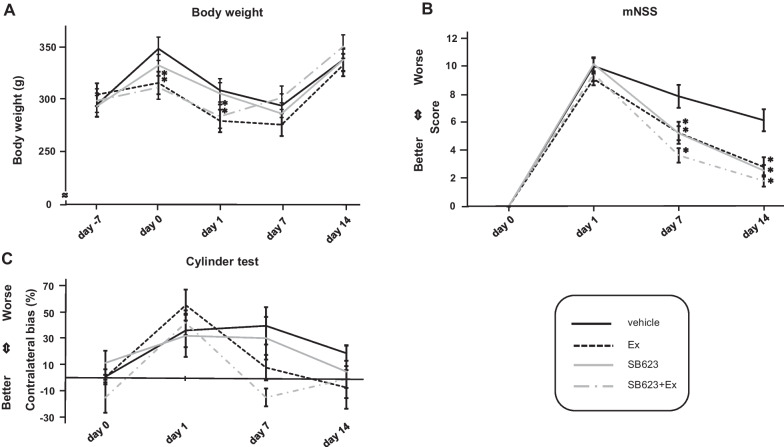
Body weight and behavioral tests for each group. **a** There was a significant difference in weight gain between the vehicle group and the voluntary exercise group from day − 7 to day 0 after MCAO. Comparing day 0 and day 1 after MCAO, body weight was decreased in all groups at day 1. ANOVA revealed a significant treatment effect with significant difference in weight loss between the voluntary exercise group (Ex and SB623 + Ex groups) and the no-exercise group (vehicle and SB623 groups) at day 1 after MCAO (*F*_(3, 31)_ = 8.7, *p* < 0.001 at day 1) (mean ± SE, ^*^p < 0.05 vs. vehicle group). Body weight decreased from day 1 to day 7, and increased from day 8 to day 14 in all groups. **b** ANOVA revealed significant treatment effects, of the Ex, SB623, and SB623 + Ex groups, respectively, in mNSS at later time points after MCAO, which significantly improved compared to that of vehicle group at day 7 and day 14 after MCAO (*F*_(3, 31)_ = 7.07, *p* < 0.001 at day 7, *F*_(3, 31)_ = 10.80, *p* < 0.001 at day 14) (mean ± SE, ^*^p < 0.05 vs. vehicle group). **c** The graph shows the results of contralateral bias in the cylinder test (vehicle group: *n* = 6; Ex group: *n* = 7; SB623 group: *n* = 5; SB623 + Ex group: *n* = 8). ANOVA revealed that there were no significant treatment effects in cylinder test from all four groups (*F*_(3, 22)_ = 1.42, *p* = 0.26). *Abbreviation*: MCAO: middle cerebral artery occlusion

All groups gained body weight from day − 7 to day 0 after MCAO. But the weight gain of the voluntary exercise groups (Ex and SB623 + Ex groups) was less than that of the no- exercise groups (vehicle and SB623 groups). ANOVA revealed significant treatment effects with post hoc Tukey’s test showing that there was a significantly different weight gain between the vehicle group and the voluntary exercise group at day 0 after MCAO (*F*_(3, 31)_ = 6.92, *p* < 0.001 at day 0) (day 0: vehicle group: 347.93 ± 6.0 g; Ex group: 315.3 ± 5.2 g; SB623 group: 332.1 ± 7.7 g; SB623 + Ex group: 311.0 ± 6.4 g, respectively). Comparing day 0 to day 1 after MCAO, body weight was decreased in all groups at day 1 after MCAO. ANOVA revealed again a significant treatment effect with post hoc Tukey’s test detecting that there was a significantly different weight loss between the voluntary exercise group and the no-exercise group at day 1 after MCAO (*F*_(3, 31)_ = 8.7, *p* < 0.001 at day 1) (day 1: vehicle group: 308.24 ± 4.84 g; Ex group: 279.08 ± 3.86 g; SB623 group: 305.21 ± 4.65 g; SB623 + Ex: 283.26 ± 5.30 g, respectively). Body weight decreased from day 1 to day 7 after MCAO, but increased from day 8 to day 14 MCAO in all groups. There was no significant difference in weight gain at day 1, 7, and 14 after MCAO between the voluntary exercise group and the no-exercise group (*F*_(3, 31)_ = 0.96, *p* = 0.43 at day 7, *F*_(3, 31)_ = 0.33, *p* = 0.80 at day 14) (day 7: vehicle group: 293.61 ± 9.80 g; Ex group: 275.45 ± 10.64 g; SB623 group: 286.00 ± 12.83 g; SB623 + Ex group: 301.17 ± 7.67 g, respectively) (day 14: vehicle group: 337.38 ± 16.20 g; Ex group: 332.36 ± 8.50 g; SB623 group: 337.24 ± 13.72 g; SB623 + Ex: 350.00 ± 7.77 g, respectively) (Fig. 3a).

### Treated stroke rats improve in mNSS score, but not in cylinder test

ANOVA revealed that there were no significant treatment effects in mNSS evaluation at day 1 after MCAO (*F*_(3, 31)_ = 1.03, *p* = 0.39 at day 1) (day 1: vehicle group: 9.78 ± 0.51; Ex group: 8.88 ± 0.41; SB623 group; 10.00 ± 0.47; SB623 + Ex group: 9.22 ± 0.47, respectively). However, ANOVA revealed significant treatment effects of the Ex, SB623, and SB623 + Ex groups, respectively, in mNSS at later time points after MCAO with significant improvement compared to the vehicle group at day 7 and day 14 after MCAO (*F*_(3, 31)_ = 7.07, *p* < 0.001 at day 7, *F*_(3, 31)_ = 10.80, *p* < 0.001 at day 14) (day 7: vehicle group: 7.67 ± 0.75; Ex group: 5.13 ± 0.72; SB623 group: 5.00 ± 0.47; SB623 + Ex group: 3.6 ± 0.47, day 14: vehicle group: 6.00 ± 0.72; Ex group: 2.75 ± 0.63; SB623 group: 2.44 ± 0.39; SB623 + Ex group: 1.78 ± 0.38, respectively). The SB623 + Ex group tended to perform better in mNSS at day 7 compared to the Ex group and the SB623 group (day 7: *p* = 0.35 vs. Ex group, *p* = 0.40 vs. SB623 group; day14: *p* = 0.65 vs. Ex group, *p* = 0.84 vs. SB623 group, respectively) (Fig. 3b).

The rats that did not touch the cylinder wall at all during the testing period were excluded from this test. The following is the number of remaining rats: vehicle group: *n* = 6; Ex group: *n* = 7; SB623 group: *n* = 5; SB623 + Ex group: *n* = 8. ANOVA revealed that there were no significant treatment effects in cylinder test from all four groups (*F*_(3, 22)_ = 1.42, *p* = 0.26) (day 0: vehicle group: 0.385 ± 5.73%; Ex group: 0.030 ± 1.90%, SB623 group: 11.00 ± 9.13%, SB623 + Ex group: − 15.4 ± 11.6%, day 1: vehicle group: 35.6 ± 12.7%; Ex group: 54.8 ± 11.8%, SB623 group: 31.5 ± 16.04%, SB623 + Ex group: 41.4 ± 9.44%, day 7: vehicle group: 39.1 ± 14.2%; Ex group: 7.36 ± 9.65%, SB623 group: 29.7 ± 16.1%, SB623 + Ex group: − 15.3 ± 6.78%, day 14: vehicle group: 18.3 ± 5.64%; Ex group: − 7.69 ± 16.1%, SB623 group: 4.32 ± 20.2%, SB623 + Ex group: − 1.31 ± 6.71%, respectively) (Fig. 3c).

### Running distance increases over time after stroke

**Fig. 4:**
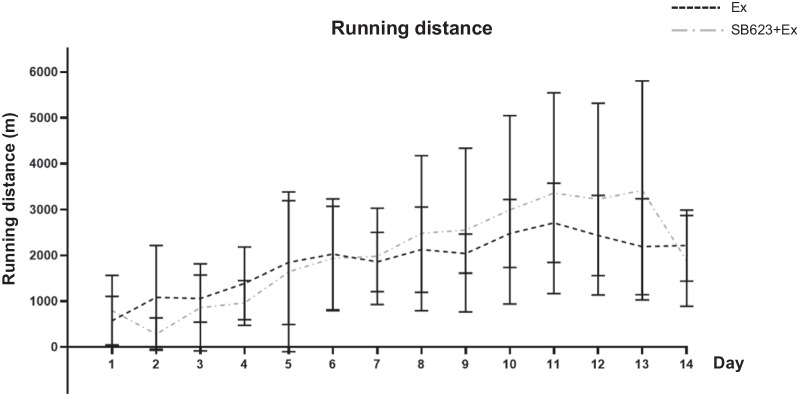
Daily running distance with running wheel after MCAO. The running distance increased day by day between the Ex group and the SB623 + Ex groups. There was no significant difference in the average running distance from day 1 to day 14 after MCAO (mean ± SD, *p* = 0.58). There was no significant difference in the average running distance at day 1, day 7, and day 14 after MCAO, respectively (mean ± SD, day 1: *p* = 0.53; day 7: *p* = 0.79; day 14: *p* = 0.48, respectively). The highest average running distance in the Ex group was 2711 m at day 11 after MCAO (mean ± SD, *p* = 0.47) and 3418 m in the SB623 + Ex group at day 13 after MCAO (mean ± SD, *p* = 0.23). *Abbreviation*: RW: running wheel; MCAO: middle cerebral artery occlusion

There were no significant treatment effects on the average running distance from day 1 to day 14 after MCAO (Ex group: 1860 ± 158 m/day; SB623 + Ex group: 2027 ± 265 m/day, *p* = 0.58). Similarly, there were no significant treatment effects on the average running distance for each post-MCAO testing period at day 1, day 7, and day 14 after MCAO, respectively (day 1: Ex group: 578 ± 528 m; SB623 + Ex group: 800 ± 767 m, *p* = 0.53, day 7: Ex group: 1857 ± 645 m; SB623 + Ex group: 1981 ± 1049 m, *p* = 0.79, day 14: Ex group: 2217 ± 777 m; SB623 + Ex group: 1881 ± 988 m, *p* = 0.48, respectively). The highest average running distance in the Ex group was 2711 m at day 11 after MCAO (Ex group: 2711 ± 868 m; SB623 + Ex group: 3357 ± 2191 m, *p* = 0.47) and 3418 m in the SB623 + Ex group at day 13 after MCAO (Ex group: 2190 ± 1047 m; SB623 + Ex group: 3418 ± 2388 m, *p* = 0.23). Running distance increased in both groups from day 2 to day 11 after MCAO (Fig. 4). And repeated measures ANOVA with post hoc Bonferroni correction did not detect significant difference between running distance and changes over time (F_(13, 195)_ = 1.21, *p* = 0.274).

### Combination therapy of SB623 intracerebral transplantation and voluntary exercise with RW most effectively reduces cerebral infarct area

**Fig. 5:**
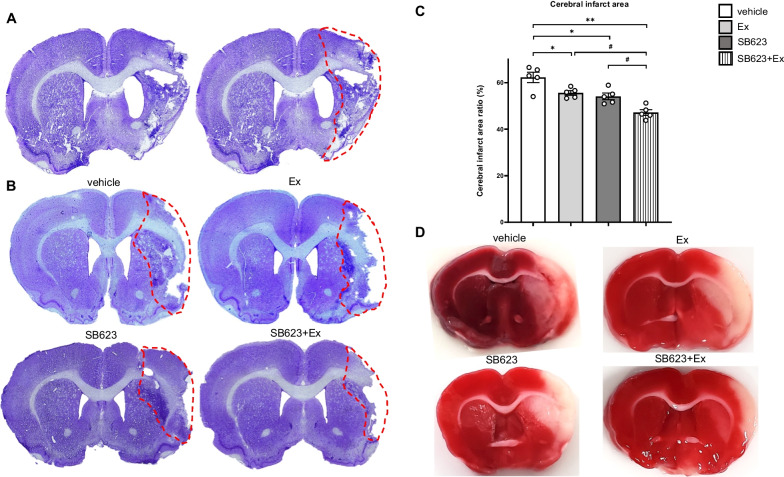
Nissl staining for cerebral infarct area and TTC staining for ischemic brain tissue in each group. **a**, **b** Cerebral infarct area is shown by Nissl staining. A **a** is representative Nissl staining for cerebral infarction area. The tract of red dotted line revealed cerebral infarct area. We calculated the cerebral infarct area ratio by the following method: Cerebral infarct area ratio = [LT – (RT – RI)] × 100/LT (%), where LT is the area of the left hemisphere, RT is the area of the right hemisphere, and RI is the infarct area in mm^2^ at the level of SVZ. **c** ANOVA detected significant treatment effects on cerebral infarct in Nissl-stained infarcted areas, which was significantly reduced in the SB623 + Ex group compared to the vehicle, Ex, and SB623 group, respectively (each group: *n* = 5, *F*_(3, 16)_ = 16.4, *p* < 0.001). The Ex group and the SB623 group showed significantly reduced infarcted areas in Nissl-stained tissue sections compared to the vehicle group (mean ± SE, ^*^p < 0.05, ^**^p < 0.01 vs. vehicle group, ^#^*p* < 0.05 vs. SB623 + Ex group). **d** Representative TTC-stained coronal sections at the level of 0.7 mm anterior from bregma are shown in each group. Infarct area appears in white, and normal tissue in red. *Abbreviation*: TTC: 2, 3, 4-triphenyltetrazolium chloride, MCAO: middle cerebral artery occlusion; SVZ: subventricular zone

Nissl staining of infarct brain tissue and cerebral infarct area is shown in Fig. 5a, b. Figure 5a and b shows representative Nissl staining for the cerebral infarction area and of all groups in our study protocol, respectively. Representative TTC staining in each group is shown in Fig. 5d (vehicle group: 64.20 ± 0.90%; Ex group: 54.46 ± 0.55%; SB623 group: 53.49 ± 1.10%; SB623 + Ex group: 44.88 ± 0.78%, respectively). ANOVA detected significant treatment effects on cerebral infarct size in Nissl-stained tissue sections, with significantly reduced infarct areas in the SB623 + Ex group compared to the vehicle, Ex, and SB623 group, respectively (*F*_(3, 16)_ = 16.4, *p* < 0.001) (vehicle group: 62.21 ± 1.97%; Ex group: 55.56 ± 0.85%; SB623 group: 54.06 ± 1.27%; SB623 + Ex group: 47.13 ± 1.11%, respectively). The Ex group and the SB623 group showed significantly reduced infarcted areas in Nissl-stained tissue sections compared to the vehicle group. However, the infarct sizes did not significantly differ between the Ex group and the SB623 group (*p* = 0.90) (Fig. 5c).

### Combination therapy of SB623 intracerebral transplantation and voluntary exercise with RW most effectively enhances endogenous neurogenesis in SVZ and DG

**Fig. 6:**
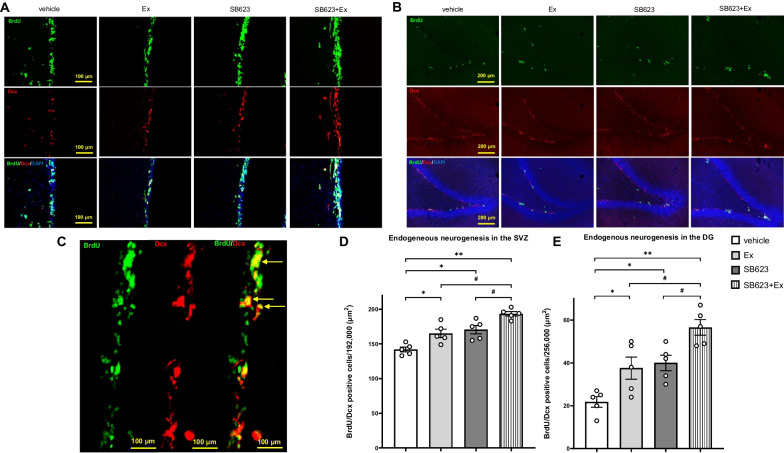
Immunofluorescent staining for endogenous neurogenesis in SVZ and DG. **a**, **b** Immunofluorescent staining for BrdU, Dcx, and DAPI shows the endogenous neurogenesis in SVZ and DG. SVZ: Scale bar = 100 μm. DG: Scale bar = 200 μm. **c** High-magnification photographs of a representative immunofluorescent staining for BrdU/Dcx double-positive cells in the SVZ (yellow arrows). Scale bar = 100 μm. **d**, **e** ANOVA detected significant treatment effects on endogenous neurogenesis with significantly increased numbers of BrdU/Dcx double-positive cells in SVZ and DG in the SB623 + Ex group compared to the vehicle, Ex, and SB623 groups, respectively (each group: *n* = 5, SVZ: *F*_(3, 16)_ = 18.9, *p* < 0.001; *F*_(3, 16)_ = 13.7, DG: *p* < 0.001). The Ex group and the SB623 group had significantly increased numbers of BrdU/Dcx double-positive cells in SVZ and DG compared to the vehicle group (SVZ: mean ± SE, ^*^p < 0.05, ^**^p < 0.01 vs. vehicle group, ^#^*p* < 0.05 vs. SB623 + Ex group; DG: ^*^p < 0.05, ^**^p < 0.01 vs. vehicle group, ^#^*p* < 0.05 vs. SB623 + Ex group). *Abbreviation*: SVZ: subventricular zone; DG: dentate gyrus; BrdU: 5-bromo2′-deoxyuridine, Dcx: Doublecortin, DAPI: 4′ 6-Diamidino-2-phenylindole

Representative photographs of immunofluorescent staining for BrdU, Dcx, and DAPI in SVZ and DG are shown in Fig. 6a–c. ANOVA detected significant treatment effects on endogenous neurogenesis with significantly increased the numbers of BrdU/Dcx double-positive cells in SVZ and DG in the SB623 + Ex group compared to in-vehicle, Ex, and SB623 groups, respectively (SVZ: *F*_(3, 16)_ = 18.9, *p* < 0.001; DG: *F*_(3, 16)_ = 13.7, *p* < 0.001) (SVZ: vehicle group: 142.00 ± 3.19/192,000 μm^2^; Ex group: 165.20 ± 5.50/192,000 μm^2^; SB623 group: 170.60 ± 5.46/192,000 μm^2^; SB623 + Ex group: 193.40 ± 2.92/192,000 μm^2^; DG: vehicle group: 21.80 ± 2.22/256,000 μm^2^; Ex group: 37.60 ± 4.64/256,000 μm^2^; SB623 group: 40.00 ± 3.20/256,000 μm^2^; SB623 + Ex group: 56.60 ± 3.28/256,000 μm^2^, respectively). The Ex group and the SB623 group significantly increased numbers of BrdU/Dcx double-positive cells in SVZ and DG compared to the vehicle group. However, the levels of endogenous neurogenesis in SVZ and DG did not significantly differ between the Ex group and the SB623 group (*p* = 0.87) (Fig. 6d and e).

### Combination therapy of SB623 intracerebral transplantation and voluntary exercise with RW most effectively enhances angiogenesis in the ischemic boundary zone

**Fig. 7:**
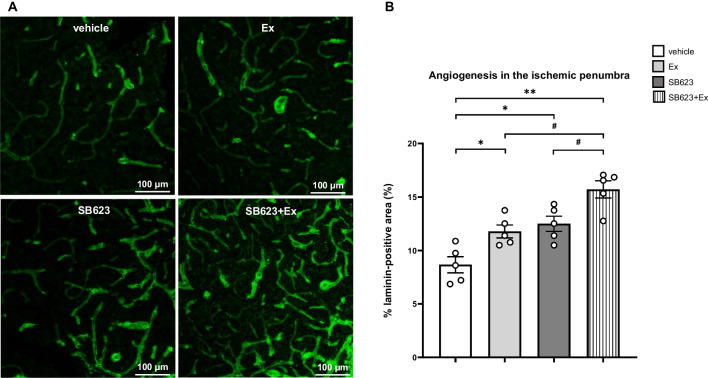
Immunofluorescent staining for angiogenesis in the IBZ. **a** Immunofluorescent staining for the laminin-positive area to evaluate angiogenesis in the IBZ. Scale bar = 100 μm. **b** ANOVA detected significant treatment effects on angiogenesis as assessed by measuring the percentage of laminin-positive area, which significantly increased in the SB623 + Ex group compared to the vehicle, Ex, and SB623 groups, respectively (each group: *n* = 5, *F*_(3, 16)_ = 14.0, *p* < 0.001). The Ex group and the SB623 group had significantly increased in laminin-positive percentage areas in the IBZ compared to the vehicle group (mean ± SE, ^*^p < 0.05, ^**^p < 0.01 vs. vehicle group, ^#^*p* < 0.05 vs. SB623 + Ex group). *Abbreviation*: IBZ: ischemic boundary zone

The laminin-positive area in the IBZ is shown in Fig. 7a. ANOVA detected significant treatment effects on angiogenesis as assessed by measuring the percentage of laminin-positive area, which significantly increased in the SB623 + Ex group compared to the vehicle, Ex, and SB623 groups, respectively (*F*_(3, 16)_ = 14.0, *p* < 0.001) (vehicle group: 8.66 ± 0.68%; Ex group: 11.79 ± 0.54%; SB623 group: 12.50 ± 0.64%: SB623 + Ex group; 15.71 ± 0.71%, respectively). The Ex group and the SB623 group had significantly increased laminin-positive percentage areas in the IBZ as compared to the vehicle group. However, the levels of laminin-positive percentage area did not significantly differ between the Ex group and the SB623 group (*p* = 0.98) (Fig. 7b).

### Robust graft survival of SB623 cells at earlier time points after transplantation

**Fig. 8:**
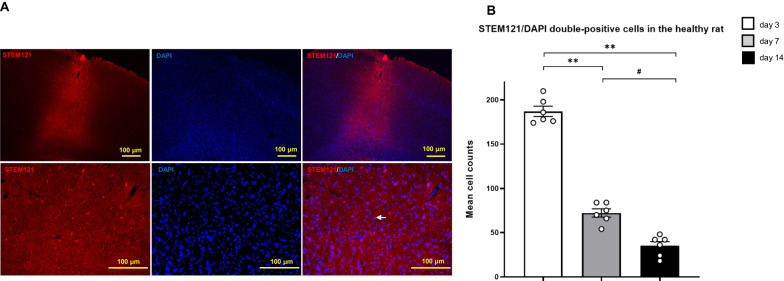
Immunofluorescent staining for the viability of SB623 cells in the healthy rat after intracerebral transplantation. **a** Immunofluorescent staining for STEM121/DAPI double-positive cells in the healthy rat shows SB623 cells at day 3 after intracerebral transplantation (white arrow) (top: low magnification, bottom: high magnification). Scale bar = 100 μm. **b** ANOVA revealed that the number of STEM121-positive cells at day 3 after transplantation was significantly higher than that at day 7 and day 14 (each group: *n* = 6) (*F*_(2, 15)_ = 264, *p* < 0.001) (mean ± SE, ^**^p < 0.01 vs. day 3 group, ^#^*p* < 0.05 vs. day 14 group). *Abbreviation*: DAPI: 4′ 6-diamidino-2-phenylindole

To examine the viability of SB623 cells in the healthy and ischemic stroke rat brain after transplantation, we performed immunofluorescence staining for STEM121 which reacts specifically with a cytoplasmic protein of human cells with nuclear staining using DAPI. Immunofluorescent staining for STEM121/DAPI double-positive cells in the healthy rat at day 3 after transplantation is shown in Fig. 8a. (A representative figure of the ischemic stroke rat at day 3 is shown in Additional file 1: Figure S3a.) ANOVA revealed that the number of STEM121-positive cells at day 3 in the healthy rat after transplantation was significantly higher than that at day 7 and day 14 (*F*_(2, 15)_ = 264, *p* < 0.001) (day 3: 187.00 ± 5.33; day 7: 72.17 ± 4.28; day 14: 35.00 ± 4.34, respectively) (Fig. 8b). ANOVA revealed that the number of STEM121-positive cells at day 3 in the ischemic stroke rat after transplantation was significantly higher than that at day 7 (*F*_(2, 5)_ = 50.2, *p* < 0.01) (day 3: 155.50 ± 3.06; day 7: 70.50 ± 12.53; day 14: 29.00 ± 2.45, respectively) (Additional file 1: Fig. S3b). However, the number of STEM121-positive cells did not significantly differ between day 7 and day 14 (*p* = 0.095). And repeated measures ANOVA with post hoc Bonferroni correction did not detect a significant difference between the healthy rat and the ischemic stroke rat (F_(1, 7)_ = 4.42, *p* = 0.080).

### Combination therapy of SB623 intracerebral transplantation and voluntary exercise with RW maximally up-regulates the expression of BDNF and VEGF in mRNA levels

**Fig. 9:**
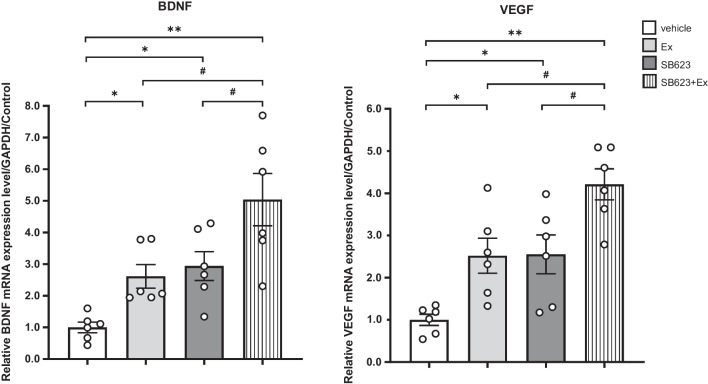
mRNA expression of BDNF and VEGF in the IBZ. ANOVA detected significant treatment effects on mRNA expressions of BDNF and VEGF, which were significantly up-regulated in the SB623 + Ex group compared to the vehicle, Ex, and SB623 groups, respectively (each group: *n* = 6, BDNF: *F*_(3, 20)_ = 10.1, *p* < 0.001, VEGF: *F*_(3, 20)_ = 13.1, *p* < 0.001) (mean ± SE, BDNF: *F*_(3, 20)_ = 10.1, *p* < 0.001, VEGF: *F*_(3, 20)_ = 13.1, *p* < 0.001). The Ex group and the SB623 group significantly up-regulated mRNA expressions of BDNF and VEGF compared to the vehicle group (^*^p < 0.05, ^**^p < 0.01 vs. vehicle group, ^#^*p* < 0.05 vs. SB623 + Ex group). *Abbreviation*: BDNF: brain-derived neurotrophic factor, VEGF: vascular endothelial growth factor, IBZ: ischemic boundary zone

To explore a mechanism possibly underlying SB623 intracerebral transplantation and voluntary exercise with RW, we assessed the mRNA expression of BDNF and VEGF using qRT-PCR. ANOVA indicated significant treatment effects on mRNA expressions of BDNF and VEGF with post hoc Tukey’s test determining that these two growth factors were significantly up-regulated in the SB623 + Ex group compared to the vehicle, Ex, and SB623 group, respectively (each group: *n* = 6) (BDNF: *F*_(3, 20)_ = 10.1, *p* < 0.001, VEGF: *F*_(3, 20)_ = 13.1, *p* < 0.001) (BDNF: vehicle group: 1.15 ± 0.19; Ex group: 2.99 ± 0.43; SB623 group: 3.36 ± 0.52; SB623 + Ex group: 5.77 ± 0.95, VEGF: vehicle group: 1.14 ± 0.15; Ex group: 2.87 ± 0.47; SB623 group: 2.91 ± 0.52; SB623 + Ex group: 4.79 ± 0.42, respectively). The Ex group and the SB623 group also had significantly up-regulated mRNA expressions of BDNF and VEGF than the vehicle group. However, these mRNA expression levels did not differ statistically between the Ex group and the SB623 group (*p* = 0.97) (Fig. 9).

### Endogenous neurogenesis in SVZ and DG negatively correlates with cerebral infarct area

**Fig. 10:**
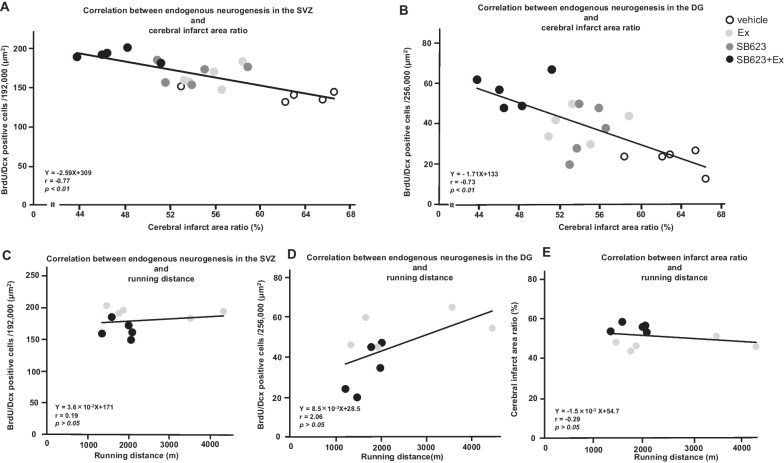
Correlational analyses between the endogenous neurogenesis, cerebral infarct area ratio, and running distance. **a**, **b** The number of BrdU/Dcx double-positive cells in the SVZ and the DG from all four groups negatively correlated with cerebral infarct area ratio (SVZ: *r* = − 0.77, *p* < 0.01; DG: *r* = − 0.73, *p* < 0.01). **c**, **d** The number of BrdU/Dcx double-positive cells and running distance in the Ex group and the SB623 + Ex group (SVZ: *r* = 0.19, *p* > 0.05; DG: *r* = 2.06 *p* > 0.05). **e** There was no significant correlation between cerebral infarct area ratio and running distance (*r* = − 0.29, *p* > 0.05). *Abbreviation*: SVZ: subventricular zone; DG: dentate gyrus; BrdU: 5-bromo2’-deoxyuridine, Dcx: Doublecortin

Pearson’s correlation analyses were performed to evaluate the relationship between the results of the immunofluorescent staining for the endogenous neurogenesis in SVZ and DG, the cerebral infarct area ratio, and the running distance. The number of BrdU/Dcx double-positive cells in SVZ and DG from all four groups negatively correlated with cerebral infarct area ratio (SVZ: *y* = − 2.59*x* + 309, *r* = − 0.77, *p* < 0.01; DG: *y* = − 1.71*x* + 133, *r* = − 0.73, *p* < 0.01) (Fig. 10a and b), indicating that endogenous neurogenesis in SVZ and DG accompanied the reduction in cerebral infarction. However, there was neither a significant correlation between the number of BrdU/Dcx double-positive cells and running distance in the Ex group and the SB623 + Ex group (SVZ: *y* = 3.6 × 10^−3^*x* + 171, *r* = 0.19, *p* > 0.05; DG: *y* = 8.5 × 10^−3^*x* + 28.5, *r* = 2.06, *p* > 0.05) (Fig. 10c and d), nor a significant correlation between cerebral infarct area ratio and running distance (*y* = − 1.5 × 10^–3^ *x* + 54.7, *r* = − 0.29, *p* > 0.05) (Fig. 10e). The correlation between running distance and mNSS, infarct area, the number of BrdU/Dcx double-positive cells, or angiogenesis was also evaluated individually. However, Pearson’s correlation analyses did not reveal any significant correlation, respectively.

## Discussion

In this study, we demonstrated that combination therapy of intracerebral transplantation of human modified bone marrow-derived MSCs, SB623 cells, and voluntary exercise with RW in a rat model of ischemic stroke achieved neurological recovery and reduced the cerebral infarct area. We also showed that this combination therapy synergistically promoted endogenous neurogenesis and increased laminin-labeled cerebral blood vessels in the IBZ. These results indicate that this combination therapy exerts strong therapeutic effects via neuroprotection, enhanced endogenous neurogenesis, and angiogenesis after ischemic insults. An up-regulation of BDNF and VEGF seems to play a role in the mechanisms of the combination therapy of SB623 cell transplantation and voluntary exercise with RW.

We used SB623 cells with which clinical trials for patients with chronic ischemic stroke (STR-01) [14] and traumatic brain injury (STEMTRA trial) [16] were performed with good clinical outcomes. In both the clinical trials, strong therapeutic potentials of SB623 cell transplantation in combination with self-exercise (rehabilitation) were implicated. Indeed, our study suggests that this combination therapy could exert synergistic therapeutic effects on a rat model of acute ischemic stroke. These results underscore the importance of performing basic research and clinical research in parallel to developing this field of science.

Few experimental studies have been conducted to evaluate the combination therapy of MSCs or other cells (e.g., neural stem cell, neural progenitor cell) transplantation and EE including RW in animal models of cerebral ischemic stroke. As far as we are aware, there are only four studies [21–23, 28]. Hicks et al. [21–23] reported three studies evaluating combination therapy of stem cell transplantation and EE in MCAO rats. Although the combination therapy of mouse SVZ-derived stem cells intracerebral transplantation showed functional recovery in the cylinder test at the very early phase (day 3) after ischemic infarct, they did not show the long-term therapeutic effects of combination therapy. Mu et al. [28] reported the combination therapy using intravenous administration of human adipose tissue-derived mesenchymal stem cells (hADMSCs) at day 2 after MCAO with voluntary exercise. Their results showed that combination therapy recovered neurological function evaluated by the cylinder test at day 21 and 42 after MCAO compared to the vehicle group. But they did not show neurological recovery at day 7 after MCAO. Our results showed that the treatment groups recovered neurological function in mNSS at day 7 and day 14 after MCAO compared to the vehicle group, but we did not observe any significant differences in the cylinder test. The discrepancy between the results of mNSS and cylinder test might lie in the fact that mNSS is influenced by not only motor performance but also other functions, such as sensation, balance, and reflexes. Additionally, exercise itself was used as one of the important treatments in this study, which might also have affected exploratory behavior which is an important factor contributing to performance in the cylinder test. This might be a reason for these confusing behavioral results. Exercise is also an important factor to consider for cell therapy in clinical settings. That is to say, the therapeutic potentials of exercise are strong and responders to exercise might be also responders to cell therapy. In the present study, however, we cannot identify the responders to each of the two treatments.

Some rats were excluded from the cylinder test because they did not engage in the test (i.e., they did not touch the wall of the cylinder at all). The following is the number of excluded rats: vehicle group: *n* = 3; Ex group: *n* = 1; SB623 group: *n* = 4; SB623 + Ex group: *n* = 1. All groups of rats did not engage in the test at day 1 after MCAO. The results of mNSS evaluation at day 1 after MCAO were average or slightly high above average within each group (vehicle group: 10.67 ± 0.27; Ex group: 10; SB623 group: 10 ± 0.61; SB623 + Ex group: 10). Also, the running distance at day 1 after MCAO was below the average running distance within each group (Ex group: 510 m; Ex + SB623 group: 557 m). It is not clear whether the cause for the lack of engagement with this exploratory test is due to paralysis or less motivation from cerebral infarct. The activity levels of most rats after MCAO might decrease in the acute phase due to post-stroke depression [48, 49]. Therefore, it is conceivable that we require to examine a follow-up study whether this cause is associated with psychological or psychiatric aspects such as post-stroke depression.

In our study design, voluntary exercise started 7 days before MCAO and restarted immediately after the completion of MCAO procedure. Although the studies of Mu and Hicks restarted voluntary exercise with EE from day 2 and day 8 after MCAO, respectively, their studies did not include pre-training before MCAO [21–23, 28]. In our study, early intervention with voluntary exercise might critically affect the improvement in behavioral results and other histological ameliorations. Therefore, the start and restart timing of exercise still remains controversial. Figure 4 shows that there is a decrease in running distance at day 14 in the SB623 + Ex group, which was not observed in the Ex group. Why the running distance decreased only in the SB623 + Ex group on that day would need further investigation. This phenomenon might be affected by the repeated BrdU intraperitoneal administration, although the reason for different effects between Ex group and SB623 + Ex group could not be explained. If we make another cohort of rats without BrdU administration, the running at day 14 might not be affected.

There are several approaches for cell therapy such as intracerebral, intravenous, and intra-arterial routes for ischemic stroke [10, 26, 42]. In our study, intracerebral transplantation was chosen. Although the advantage of intracerebral transplantation is the accurate delivery of stem cells to the vicinity of the lesioned brain, this approach is more invasive compared to intravenous and intra-arterial transplantations. To achieve strong therapeutic effects, patients with chronic ischemic stroke (STR-01) [14] and traumatic brain injury (STEMTRA trial) [16] received intracerebral transplantation of SB623 cells. In both clinical trials, intracerebral SB623 administration seemed safe and well-tolerated. In addition, some basic research studies revealed the superiority of intracerebral transplantation compared to intravenous transplantation of MSCs or neural progenitor cells in a rat model of ischemic stroke [50, 51].

The four studies did not reveal a significant reduction in cerebral infarct volumes in treatment groups, compared to the control group [21–23, 28]. Yasuhara et al*.* reported that intracerebral transplantation of SB623 cells in a rat model of ischemic stroke at the chronic phase reduced ischemic cell loss in the peri-infarct area. Tate et al. reported that SB623 cells secreted the neurotrophic factors such as BDNF and VEGF in an in *vitro* model of ischemia. Our results revealed the up-regulation of BDNF and VEGF after intracerebral transplantation of SB623 cells and voluntary exercise. Particularly, combination therapy of SB623 cells transplantation and voluntary exercise showed significantly higher up-regulation of both factors. This might be a critical reason behind the strong therapeutic effects of the combination therapy.

It remains controversial whether the effect of voluntary exercise reduces cerebral infarct volumes [20, 52, 53]. Marin et al. [20] reported that voluntary exercise with RW did not reduce infarct volumes in a rat model of ischemic stroke. On the other hand, Zhang et al*.* revealed the reduction in infarct volumes with suppressed apoptotic cell loss by voluntary exercise through increased secretion of BDNF. Our study revealed that voluntary exercise by itself significantly showed therapeutic effects in a rat model for ischemic stroke. Similarly, SB623 cell transplantation singly showed therapeutic potentials. However, the combination therapy of cell transplantation and exercise showed the best treatment effects. In consideration of clinical settings, rehabilitation is essential for stroke patients to achieve good functional recovery [54, 55].

In our study, the neuroprotective potentials of physical rehabilitation are at least partly involved in the groups that had the possibility to voluntary exercise. At the same time, these groups also had to have voluntary motivation, which might be important in rehabilitation also in clinical settings. Further studies are needed to reveal the difference between voluntary vs. forced exercises in some pathological conditions. In addition, we performed intracerebral transplantation of SB623 cells just one day after MCAO. This time course might be different from the clinical practice. However, at the same time, our previous study reported that MSC transplantation at 24 h after stroke provided the strongest therapeutic effects in a rat model of ischemic stroke [42]. The present study aimed to establish the evidence for the combination therapy of SB623 cell transplantation and voluntary exercise as rehabilitation. In the future, chronic phase study should be planned in consideration of the clinical application. Furthermore, in clinical studies of cell transplantation, study designs and control settings in terms of rehabilitation critically affect the results of the studies and should be considered deliberately.

Similarly, four studies have not revealed neurogenesis in the SVZ and the DG [21–23, 28]. Komitova et al*.* [18] reported that EE with RW promoted cell proliferation and neurogenesis in the SVZ at the chronic phase of ischemic stroke and altered lesion-induced progenitor cell differentiation by increasing ipsilateral astrocytes in the hippocampus [56]. It was considered as possible mechanisms that the efficacy of voluntary exercise contributed to growth factors such as neurotrophin-3 and BDNF in the SVZ [18] and hippocampus [56, 57]. Bao et al. [58] reported that human bone marrow-derived MSCs (hBMSCs) intracerebral transplantation promoted the neurogenesis in the SVZ and SGZ at day 21 after MCAO. They revealed higher levels of BDNF and VEGF in the ipsilateral ischemic boundary zone by analyzing ELISA and qRT-PCR. In our results, the expression levels of BDNF and VEGF were significantly higher after combination therapy of SB623 cells and voluntary exercise than after each single therapy at day 14 after MCAO.

Regarding angiogenesis, it was reported that combination therapies of MSCs or other cell transplantation and voluntary exercise had no significant angiogenic effects, compared to the control group and to the single therapy group [21–23, 28]. In several studies, however, MSCs transplantation therapy and exercise therapy enhanced angiogenesis in the ischemic penumbra [58–64]. Notably, as for voluntary exercise with RW, Shoyaib et al*.* revealed increased VEGF receptor 2 which is deemed the primary receptor in transducing angiogenesis-associated effects of VEGF compared to the control group [65]. These previous studies may help to explain our result about enhancing angiogenesis by combination therapy.

Our study analyzed the correlation between endogenous neurogenesis, cerebral infarct area, and running distance with RW. To the best of our knowledge, there have been no studies that have measured running distance with RW in so much detail as our study because there are few previous studies about combination therapy. We hypothesized that running distance might correlate positively with the endogenous neurogenesis in the SVZ and the DG and negatively with cerebral infarct area. However, our results revealed that there were no significant relationships between running distance and endogenous neurogenesis or cerebral infarct area. On the other hand, there was a significant negative correlation between cerebral infarct area and endogenous neurogenesis in the SVZ and DG. This result might be due to the rescued stem cell renewal in the SVZ by voluntary exercise, the increased neural stem/progenitor cell pool in the SVZ and DG, the increase in the recruitment of the generated neural precursors to the damaged neural tissue [18], and neurotrophic factor secretion including BDNF and VEGF by SB623 cells [11, 33]. The increased secretion of BDNF and VEGF might be from the transplanted SB623 cells and surrounding host tissues, resulting in the enhancement of neurogenesis at the SVZ and DG. By the same token, increased BDNF and VEGF might directly alleviate the neurological damage after ischemic insult. In addition, we showed that a few BrdU/Dcx double-positive cells migrated from the SVZ to the injury side (Additional file 1: Fig. S4), which might be involved in the neurological recovery and infarct area reduction. These effects are important to promote plasticity and regeneration in ischemic stroke brains.

## Limitations

Some limitations of this study should be acknowledged. First, only intracerebral transplantation, but not intra-arterial/intravenous transplantation, was used in this study. We attempted to approximate the clinical studies using SB623 cells in this basic research study [14, 16].

Second, the viability of SB623 cells in vivo at day 3 was approximately 0.046% (185 surviving cells after injection of a total number of 4.0 × 10^5^ cells). Our study showed that the viability decreased over time, but these results revealed very low graft survival even at day 3. These results might be due to technical problems, including the external leakage of cells by intracerebral transplantation, the technical difficulty of immunofluorescent staining for STEM121, and the immunomodulation due to xenotransplantation. These technical issues might hinder the evaluation of the exact viability and cellular behavior of SB623 cells.

Third, the results of behavioral tests indicated only acute synergistic effects. There were no differences in neurological functions between SB623 + Ex group and SB623 group or Ex group. Synergistic therapeutic effects were also observed in the infarction area and neurogenesis in the SVZ and DG. The non-correlated results of behavioral evaluations might be related to the natural recovery of rats after MCAO, the variance of behavioral results, or the types of behavioral evaluations. Cylinder test is meant to evaluate the motor function itself and mNSS can evaluate not only motor function but also various functions. Our results might suggest that the beneficial effects of voluntary exercise and SB623 implantation in the acute phase might be shown mainly in non-motor function, but not in motor function itself. This hypothesis might be reasonable considering small synergistic effects observed in the cylinder test. Additionally, in the cylinder test some rats were inactive with few forelimb touches. This might be due to the psychological influence of the experiment.

Fourth, the total number of evaluated rats was small. We performed histological evaluations on 5–6 rats in each group, which were randomly selected by a blinded examiner. We confirmed all data for normal distribution using Shapiro–Wilk normality test.

Fifth, we did not include any female test subjects in this study. The recommendations of the National Institutes of Health (NIH) in 2016 for experimental design reported that researchers should consider if and how the female estrous cycle is relevant for experimental design [66]. However, estrogen was known to affect the ischemic stroke [67, 68]. In this study, we would like to omit the influence of sex differences. Additionally, in terms of animal protection, we might need double rats to perform behavioral and histological evaluations if both genders were evaluated. In the future, we need to perform experiments with male and female animals after obtaining enough amount of SB623 cells to investigate potential sex differences.

Lastly, our study did not fully examine the complex therapeutic mechanisms of combination therapy of cell transplantation and exercise. Although we detected the up-regulation of the mRNA expression level of BDNF and VEGF in the combination therapy, we did not assess the protein level of BDNF and VEGF using ELISA and western blot method. Additionally, direct probing of these trophic factors signaling pathways as well as comprehensive mechanistic analyses should be pursued in future studies.

## Conclusions

This study demonstrates that the combination therapy of SB623 intracerebral transplantation and voluntary exercise with RW in a rat model of ischemic stroke achieves neurological recovery. This combination therapy synergistically reduces cerebral infarct area, promotes endogenous neurogenesis in the SVZ and the DG, and promotes angiogenesis in the IBZ compared to the sole SB623 cell transplantation group and the sole voluntary exercise group. Our results suggest that this combination therapy exerts synergistic therapeutic effects on a rat model of ischemic stroke. In consideration of the clinical application, rehabilitation is essential to achieve enhanced therapeutic effects of cell therapy.

## Supplementary Information

**Additional file 1**. **Figure S1**: Illustration of counting BrdU/Dcx double-positive cells in the SZV and DG. (a, b) Brdu/Dcx double-positive cells were counted in 16 areas (4 areas × 2 sections × 2 hemispheres) for the SVZ, and in 32 areas (8 areas × 2 sections × 2 hemispheres) for the DG in each rat. Abbreviation: SVZ: subventricular zone; DG: dentate gyrus; BrdU: 5-bromo2’-deoxyuridine, Dcx: Doublecortin. **Figure S2**: Immunofluorescent staining for angiogenesis in the ischemic boundary zone. Representative lower-magnification photographs of laminin-positive area in SB623+Ex group are shown. We measured the area of laminin-positive structures of randomly captured images (500 × 500 μm square) in the ischemic cortex from two different IBZ slices (0 and 0.5 mm anterior to the bregma) (left: low magnification, right upper and right lower: high magnification). scale bar = 100 μm. Abbreviation: IBZ: ischemic boundary zone. **Figure S3**: Immunofluorescent staining for the viability of SB623 cells in the ischemic stroke rat after intracerebral transplantation. (a) Immunofluorescent staining for STEM121/DAPI double-positive cells shows SB623 cells at day 3 in the ischemic stroke rat after intracerebral transplantation (white arrow) (upper: low magnification, lower: high magnification). scale bar = 100 μm. (b) The number of STEM121-positive cells in the ischemic stroke rat at day 3 after transplantation tended to be higher than that at day 7(each group: n = 2) (F (2, 5) = 50.2, p < 0.01) (mean ± SE, *p < 0.05, **p < 0.01 vs. day 3). Abbreviation: DAPI: 4’ 6-diamidino-2-phenylindole. **Figure S4**: Migration of transplanted SB623 cells in SB623+Ex group. Immunofluorescent staining for BrdU (A), Dcx (B), and BrdU/Dcx (C) in the SVZ shows SB623 cells migrated toward the injury side. Yellow arrows show the most migrated BrdU/Dcx double-positive cell. scale bar = 100 μm. Abbreviation: SVZ: subventricular zone; BrdU: 5-bromo2’-deoxyuridine, Dcx: Doublecortin.

## Data Availability

All data generated or analyzed during this study are included in this published article.

## Abbreviations

ADMSCs: Adipose tissue-derived mesenchymal stem cell
BDNF: Brain-derived neurotrophic factor
BMSCs: Bone marrow mesenchymal stromal cell
BrdU: 5-bromo2’-deoxyuridine
DAPI: 4′ 6-diamidino-2-phenylindole
Dcx: Doublecortin
DG: Dentate gyrus
DMEM: Dulbecco’s modified Eagle’s medium
ECA: External carotid artery
EE: Enriched environment
GAPDH: Glyceraldelyde-3-phosphate dehydrogenase
hASCs: Human adipose stem cells
hESC: Human embryonic stem cell
ICA: Internal carotid artery
IGF-1: Insulin-like growth factor-1
IBZ: Ischemic boundary zone
MCAO: Middle cerebral artery occlusion
MSCs: Mesenchymal stromal cells
mNSS: Modified neurological severity score
PBS: Phosphate-buffered saline
qRT-PCR: Quantitative real-time PCR
RW: Running wheel
SGZ: Subgranular cell zone
SVZ: Subventricular zone
VEGF: Vascular endothelial growth factor

## References

[1] Meschia JF, Brott T (2018). Ischaemic stroke. Eur J Neurol.

[2] Feigin VL, Norrving B, Mensah GA (2017). Global burden of stroke. Circ Res.

[3] Emberson J, Lees KR, Lyden P, Blackwell L, Albers G, Bluhmki E, Brott T (2014). Effect of treatment delay, age, and stroke severity on the effects of intravenous thrombolysis with alteplase for acute ischaemic stroke: a meta-analysis of individual patient data from randomised trials. Lancet.

[4] Nogueira RG, Jadhav AP, Haussen DC, Bonafe A, Budzik RF, Bhuva P (2018). Thrombectomy 6 to 24 h after stroke with a mismatch between deficit and infarct. N Engl J Med.

[5] Grossman AW, Broderick JP (2013). Advances and challenges in treatment and prevention of ischemic stroke. Ann Neurol.

[6] Daadi MM, Hu S, Klausner J, Li Z, Sofilos M, Sun G (2013). Imaging neural stem cell graft-induced structural repair in stroke. Cell Transpl.

[7] Ryu S, Lee SH, Kim SU, Yoon BW (2016). Human neural stem cells promote proliferation of endogenous neural stem cells and enhance angiogenesis in ischemic rat brain. Neural Regen Res.

[8] Horie N, Hiu T, Nagata I (2015). Stem cell transplantation enhances endogenous brain repair after experimental stroke. Neurol Med Chir (Tokyo).

[9] Oshita J, Okazaki T, Mitsuhara T, Imura T, Nakagawa K, Otsuka T (2020). Early transplantation of human cranial bone-derived mesenchymal stem cells enhances functional recovery in ischemic stroke model rats. Neurol Med Chir (Tokyo).

[10] Yasuhara T, Matsukawa N, Hara K, Maki M, Ali MM, Yu SJ (2009). Notch-induced rat and human bone marrow stromal cell grafts reduce ischemic cell loss and ameliorate behavioral deficits in chronic stroke animals. Stem Cells Dev.

[11] Tate CC, Fonck C, McGrogan M, Case CC (2010). Human mesenchymal stromal cells and their derivative, SB623 cells, rescue neural cells via trophic support following in vitro ischemia. Cell Transplant.

[12] Dao M, Tate CC, McGrogan M, Case CC (2013). Comparing the angiogenic potency of naïve marrow stromal cells and Notch-transfected marrow stromal cells. J Transl Med.

[13] Tajiri N, Kaneko Y, Shinozuka K, Ishikawa H, Yankee E, McGrogan M (2013). Stem cell recruitment of newly formed host cells via a successful seduction? Filling the gap between neurogenic niche and injured brain site. PLoS One..

[14] Steinberg GK, Kondziolka D, Wechsler LR, Lunsford LD, Coburn ML, Billigen JB (2016). Clinical outcomes of transplanted modified bone marrow-derived mesenchymal stem cells in stroke: a phase 1/2a study. Stroke.

[15] Steinberg GK, Kondziolka D, Wechsler LR, Lunsford LD, Kim AS, Johnson JN (2018). Two-year safety and clinical outcomes in chronic ischemic stroke patients after implantation of modified bone marrow-derived mesenchymal stem cells (SB623): a phase 1/2a study. J Neurosurg.

[16] Kawabori M, Weintraub AH, Imai H, Zinkevych L, McAllister P, Steinberg GK (2021). Cell therapy for chronic TBI: interim analysis of the randomized controlled STEMTRA trial. Neurology.

[17] Langhorne P, Bernhardt J, Kwakkel G (2011). Stroke rehabilitation. Lancet.

[18] Komitova M, Mattsson B, Johansson BB, Eriksson PS (2005). Enriched environment increases neural stem/progenitor cell proliferation and neurogenesis in the subventricular zone of stroke-lesioned adult rats. Stroke.

[19] Komitova M, Zhao LR, Gido G, Johansson BB, Eriksson P (2005). Postischemic exercise attenuates whereas enriched environment has certain enhancing effects on lesion-induced subventricular zone activation in the adult rat. Eur J Neurosci.

[20] Marin R, Williams A, Hale S, Burge B, Mense M, Bauman R (2003). The effect of voluntary exercise exposure on histological and neurobehavioral outcomes after ischemic brain injury in the rat. Physiol Behav.

[21] Hicks AU, Hewlett K, Windle V, Chernenko G, Ploughman M, Jolkkonen J (2007). Enriched environment enhances transplanted subventricular zone stem cell migration and functional recovery after stroke. Neuroscience.

[22] Hicks AU, MacLellan CL, Chernenko GA, Corbett D (2008). Long-term assessment of enriched housing and subventricular zone derived cell transplantation after focal ischemia in rats. Brain Res.

[23] Hicks AU, Lappalainen RS, Narkilahti S, Suuronen R, Corbett D, Sivenius J (2009). Transplantation of human embryonic stem cell-derived neural precursor cells and enriched environment after cortical stroke in rats: cell survival and functional recovery. Eur J Neurosci.

[24] Seo JH, Kim H, Park ES, Lee JE, Kim DW, Kim HO (2013). Environmental enrichment synergistically improves functional recovery by transplanted adipose stem cells in chronic hypoxic-ischemic brain injury. Cell Transpl.

[25] Cho SR, Suh H, Yu JH, Kim HH, Seo JH, Seo CH (2016). Astroglial activation by an enriched environment after transplantation of mesenchymal stem cells enhances angiogenesis after hypoxic-ischemic brain injury. Int J Mol Sci.

[26] Sasaki Y, Sasaki M, Kataoka-Sasaki Y, Nakazaki M, Nagahama H, Suzuki J (2016). Synergic effects of rehabilitation and intravenous infusion of mesenchymal stem cells after stroke in rats. Phys Ther.

[27] Zhao K, Li R, Bi S, Li Y, Liu L, Jia YL (2018). Combination of mild therapeutic hypothermia and adipose-derived stem cells for ischemic brain injury. Neural Regen Res.

[28] Mu J, Bakreen A, Juntunen M, Korhonen P, Oinonen E, Cui L (2019). Combined adipose tissue-derived mesenchymal stem cell therapy and rehabilitation in experimental stroke. Front Neurol.

[29] Savitz SI, Cramer SC, Wechsler L, STEPS 3 Consortium (2014). Stem cells as an emerging paradigm in stroke 3: enhancing the development of clinical trials. Stroke..

[30] Boltze J, Modo MM, Mays RW, Taguchi A, Jolkkonen J, Savitz SI (2019). Stem cells as an emerging paradigm in stroke 4: advancing and accelerating preclinical research. Stroke.

[31] Dao MA, Tate CC, Aizman I, MacGrogan M, Case CC (2011). Comparing the immunosuppressive potency of naïve marrow stromal cells and Notch-transfected marrow stromal cells. J Neuroinflammation.

[32] Dezawa M, Kanno H, Hoshino M, Cho H, Matsumoto N, Itokazu Y (2004). Specific induction of neuronal cells from bone marrow stromal cells and application for autologous transplantation. J Clin Investig.

[33] Aizman I, Tate CC, McGrogan M, Case CC (2009). Extracellular matrix produced by bone marrow stromal cells and by their derivative, SB623 cells, supports neural cell growth. J Neurosci Res.

[34] Tate CC, Chou VP, Campos C, Moalem AS, Di Monte DA, McGrogan M (2017). Mesenchymal stromal SB623 cell implantation mitigates nigrostriatal dopaminergic damage in a mouse model of Parkinson's disease. J Tissue Eng Regen Med.

[35] Morimoto J, Yasuhara T, Kameda M, Umakoshi M, Kin I, Kuwahara K (2018). Electrical stimulation enhances migratory ability of transplanted bone marrow stromal cells in a rodent ischemic stroke model. Cell Physiol Biochem.

[36] Shen LH, Li Y, Chen J, Zhang J, Vanguri P, Borneman J (2006). Intracarotid transplantation of bone marrow stromal cells increases axon-myelin remodeling after stroke. Neuroscience.

[37] Wakabayashi K, Nagai A, Sheikh AM, Shiota Y, Narantuya D, Watanabe T (2010). Transplantation of human mesenchymal stem cells promotes functional improvement and increased expression of neurotrophic factors in a rat focal cerebral ischemia model. J Neurosci Res.

[38] Schallert T, Fleming SM, Leasure JL, Tillerson JL, Bland ST (2000). CNS plasticity and assessment of forelimb sensorimotor outcome in unilateral rat models of stroke, cortical ablation, parkinsonism and spinal cord injury. Neuropharmacology.

[39] Hua Y, Schallert T, Keep RF, Wu J, Hoff JT, Xi G (2002). Behavioral tests after intracerebral hemorrhage in the rat. Stroke.

[40] Schaar KL, Brenneman MM, Savitz SI (2010). Functional assessments in the rodent stroke model. Exp Transl Stroke Med.

[41] Kuwahara K, Sasaki T, Yasuhara T, Kameda M, Okazaki Y, Hosomoto K (2020). Long-term continuous cervical spinal cord stimulation exerts neuroprotective effects in experimental Parkinson's disease. Front Aging Neurosci.

[42] Toyoshima A, Yasuhara T, Kameda M, Morimoto J, Takeuchi H, Wang F (2015). Intra-arterial transplantation of allogeneic mesenchymal stem cells mounts neuroprotective effects in a transient ischemic stroke model in rats: analyses of therapeutic time window and its mechanisms. PLoS One..

[43] Sato K, Kameda M, Yasuhara T, Agari T, Baba T, Wang F (2013). Neuroprotective effects of liraglutide for stroke model of rats. Int J Mol Sci.

[44] Yasuhara T, Hara K, Maki M, Matsukawa N, Fujino H, Date I (2007). Lack of exercise, via hindlimb suspension, impedes endogenous neurogenesis. Neuroscience.

[45] Greisen MH, Altar CA, Bolwig TG, Whitehead R, Wortwein G (2005). Increased adult hippocampal brain-derived neurotrophic factor and normal levels of neurogenesis in maternal separation rats. J Neurosci Res.

[46] Kin K, Yasuhara T, Kameda M, Agari T, Sasaki T, Morimoto J (2017). Hippocampal neurogenesis of Wistar Kyoto rats is congenitally impaired and correlated with stress resistance. Behav Brain Res..

[47] Livak KJ, Schmittgen TD (2001). Analysis of relative gene expression data using real-time quantitative PCR and the 2^-ΔΔCT^ Method. Methods.

[48] Tao X, Yang W, Zhu S, Que R, Liu C, Fan T (2019). Models of poststroke depression and assessments of core depressive symptoms in rodents: How to choose?. Exp Neurol..

[49] Lin R, Lang M, Heinsinger N, Stricsek G, Zhang J, Iozzo R (2018). Stepwise impairment of neural stem cell proliferation and neurogenesis concomitant with disruption of blood-brain barrier in recurrent ischemic stroke. Neurobiol Dis..

[50] Noh JE, Oh SH, Park IH, Song J (2020). Intracerebral transplants of GMP-grade human umbilical cord-derived mesenchymal stromal cells effectively treat subacute-phase ischemic stroke in a rodent model. Front Cell Neurosci..

[51] Jin K, Sun Y, Xie L, Park IH (2005). Comparison of ischemia-directed migration of neural precursor cells after intrastriatal, intraventricular, or intravenous transplantation in the rat. Neurobiol Dis..

[52] Kerr AL, Curtis MT, Dominguez M, Viola V (2018). Poststroke exercise is as effective as skilled rehabilitation: effects in young and aged mice. Behav Neurosci.

[53] Nemchek V, Haan EM, Mavros R, Macuiba A, Kerr AL (2021). Voluntary exercise ameliorates the good limb training effect in a mouse model of stroke. Exp Brain Res.

[54] Craig LE, Bernhardt J, Langhorne P, Wu O (2010). Early mobilization after stroke: an example of an individual patient data meta-analysis of a complex intervention. Stroke.

[55] Sundseth A, Thommessen B, Ronning OM (2012). Outcome after mobilization within 24 h of acute stroke: a randomized controlled trial. Stroke..

[56] Komitova M, Perfilieva E, Mattsson B, Eriksson PS, Johansson BB (2002). Effect of cortical ischemia and postischemic environmental enrichment on hippocampal cell genesis and differentiation in the adult rat. J Cereb Blood Flow Metab.

[57] Ickes BR, Pham TM, Sanders LA, Albeck DS, Mohammed AH, Granholm AC (2000). Long-term environmental enrichment leads to regional increases in neurotrophin levels in rat brain. Exp Neurol.

[58] Bao X, Wei J, Feng M, Lu S, Li G, Dou W (2011). Transplantation of human bone marrow-derived mesenchymal stem cells promotes behavioral recovery and endogenous neurogenesis after cerebral ischemia in rats. Brain Res..

[59] Bao X, Feng M, Wei J, Han Q, Zhao H, Li G (2011). Transplantation of Flk-1+ human bone marrow-derived mesenchymal stem cells promotes angiogenesis and neurogenesis after cerebral ischemia in rats. Eur J Neurosci.

[60] Li J, Zhang Q, Wang W, Lin F, Wang S, Zhao J (2021). Mesenchymal stem cell therapy for ischemic stroke: A look into treatment mechanism and therapeutic potential. J Neurol.

[61] Zacharek A, Shehadah A, Chen J, Cui X, Roberts C, Lu M (2010). Comparison of bone marrow stromal cells derived from stroke and normal rats for stroke treatment. Stroke.

[62] Chen Z, Hu Q, Xie Q, Wu S, Pang Q, Liu M (2019). Effects of treadmill exercise on motor and cognitive function recovery of MCAO mice through the caveolin-1/VEGF signaling pathway in ischemic penumbra. Neurochem Res.

[63] Pianta S, Lee JY, Tuazon JP, Castelli V, Mantohac LM, Tajiri N (2019). A short bout of exercise prior to stroke improves functional outcomes by enhancing angiogenesis. Neuromolecular Med.

[64] Chen X, Zhang X, Liao W, Wan Q (2017). Effect of physical and social components of enriched environment on astrocytes proliferation in rats after cerebral ischemia/reperfusion injury. Neurochem Res.

[65] Al Shoyaib A, Alamri FF, Biggers A, Karamyan ST, Arumugam TV, Ahsan F (2021). Delayed exercise-induced upregulation of angiogenic proteins and recovery of motor function after photothrombotic stroke in mice. Neuroscience.

[66] National Institutes of Health. Consideration of sex as a biological variable in NIH-funded research. 2015;NOT-OD-15-102.

[67] Liu F, McCullough LD (2011). Middle cerebral artery occlusion model in rodents: methods and potential pitfalls. J Biomed Biotechnol..

[68] Céspedes Rubio ÁE, Pérez-Alvarez MJ, Chala CL, Wandosell F (2018). Sex steroid hormones as neuroprotective elements in ischemia models. J Endocrinol.

