# Correction: Synergistic therapeutic effects of intracerebral transplantation of human modified bone marrow-derived stromal cells (SB623) and voluntary exercise with running wheel in a rat model of ischemic stroke

**DOI:** 10.1186/s13287-023-03362-z

**Published:** 2023-05-08

**Authors:** Satoru Yabuno, Takao Yasuhara, Takayuki Nagase, Satoshi Kawauchi, Chiaki Sugahara, Yosuke Okazaki, Kakeru Hosomoto, Susumu Sasada, Tatsuya Sasaki, Naoki Tajiri, Cesar V. Borlongan, Isao Date

**Affiliations:** 1 Department of Neurological Surgery, Okayama University Graduate School of Medicine, Dentistry and Pharmaceutical Sciences, 2-5-1 Shikata-cho, Kita-ku, Okayama, 700-8558 Japan; 2 Department of Neurophysiology and Brain Science, Nagoya City University Graduate School of Medical Sciences and Medical School, Nagoya, Japan; 3 Department of Neurosurgery and Brain Repair, Morsani College of Medicine, University of South Florida, Tampa, FL USA

**Correction: Stem Cell Research & Therapy (2023) 14:10** https://doi.org/10.1186/s13287-023-03236-4

The authors wish to amend affiliation #1 in the original article [1] to the following:

Department of Neurological Surgery, Okayama University Graduate School of Medicine, Dentistry and Pharmaceutical Sciences, 2-5-1 Shikata-cho, Kita-ku, Okayama, 700-8558, Japan.
