# Supplementary figures and images for: Synergistic therapeutic effects of intracerebral transplantation of human modified bone marrow-derived stromal cells (SB623) and voluntary exercise with running wheel in a rat model of ischemic stroke
Source: Stem Cell Res Ther. 2023 Jan 24;14:10. doi: 10.1186/s13287-023-03236-4 (PMC9872315; doi:10.1186/s13287-023-03236-4)

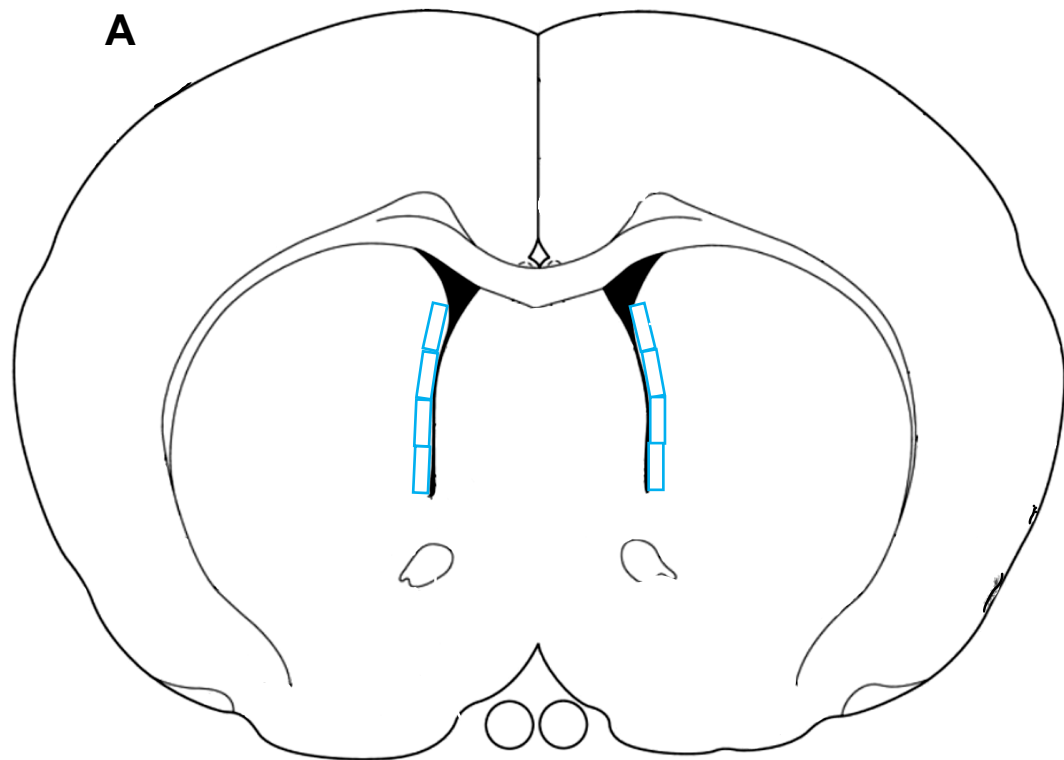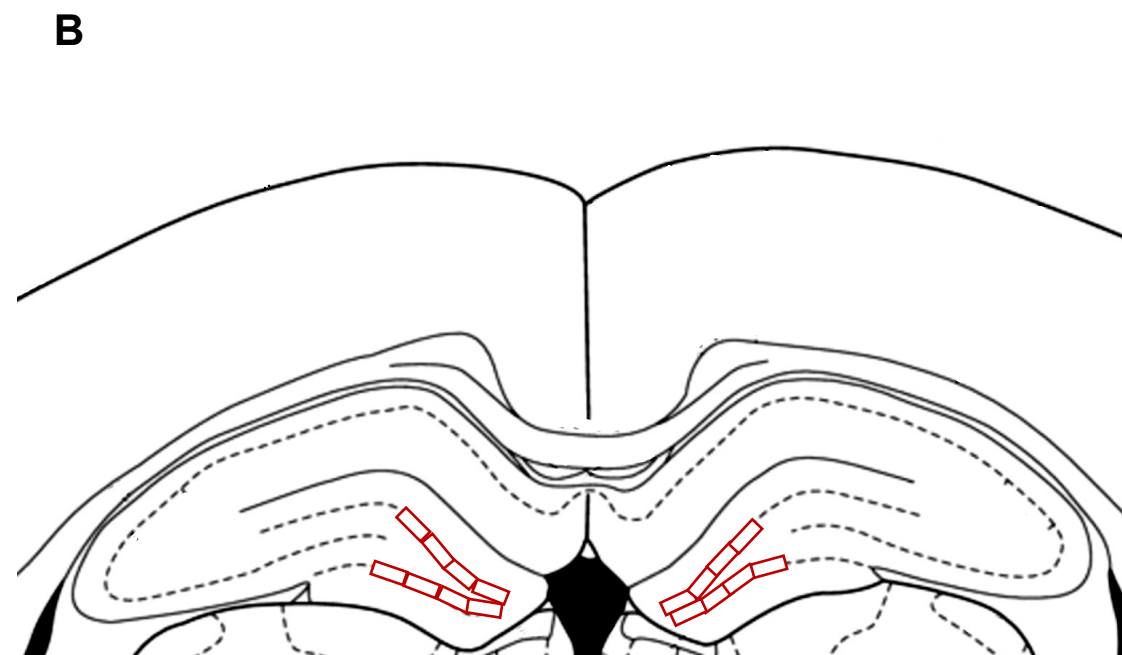

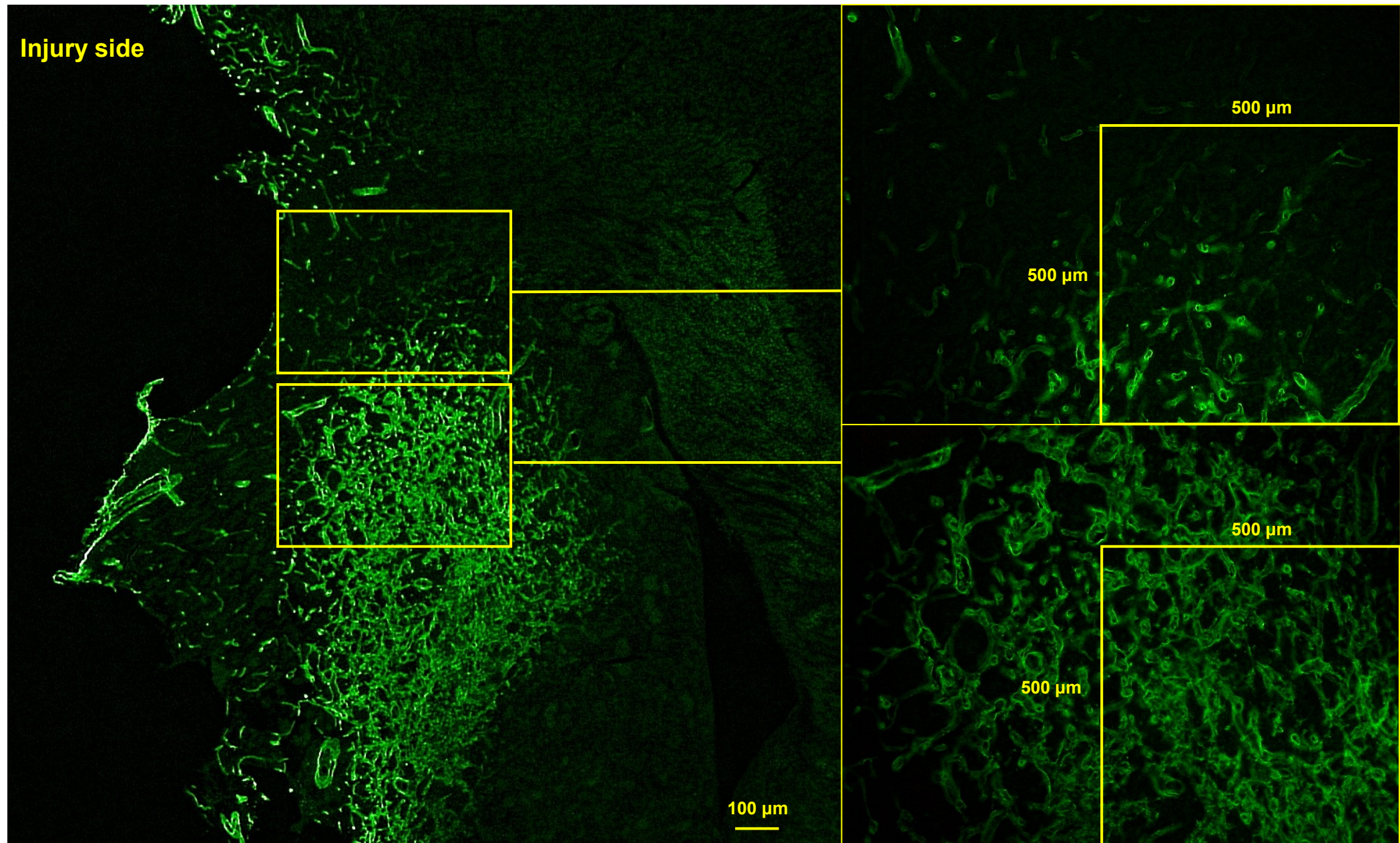

**A**

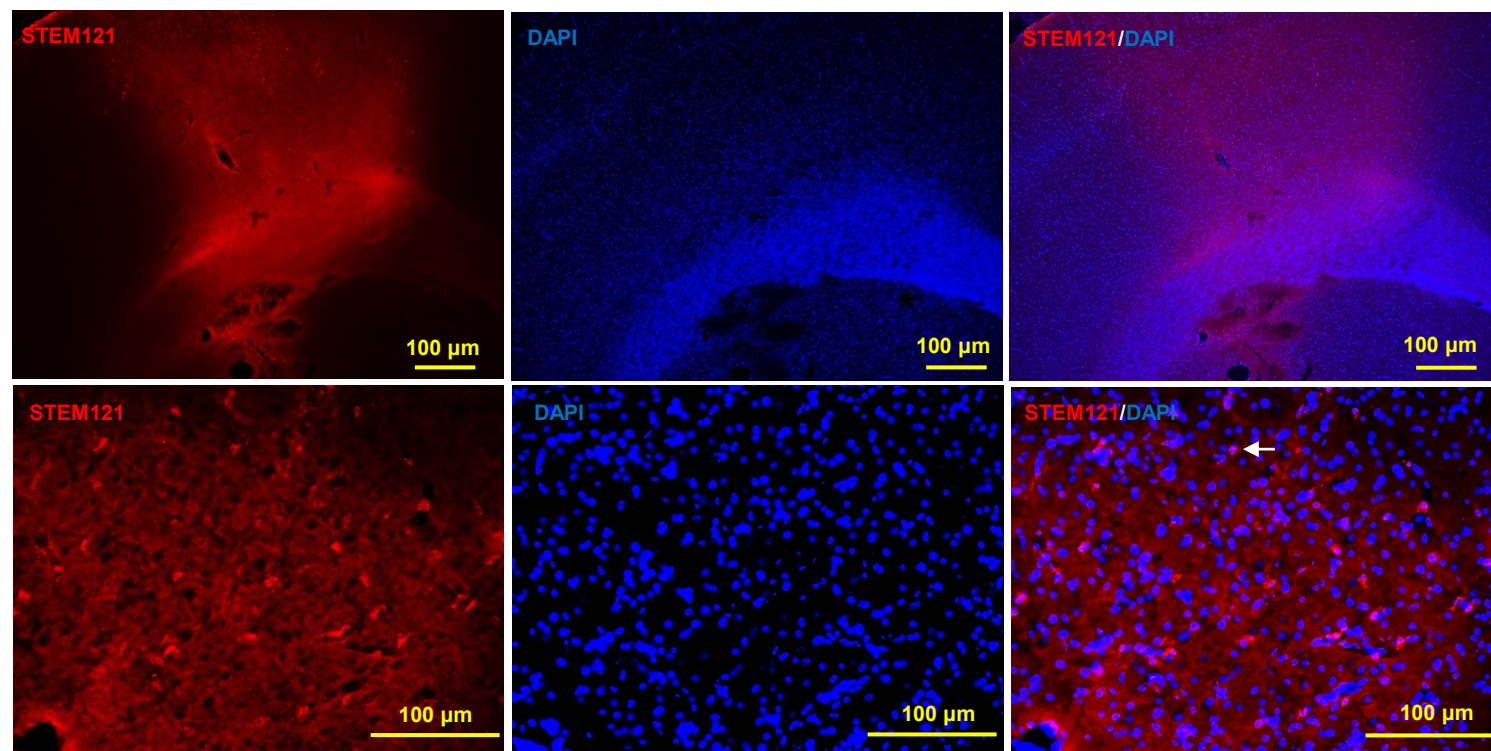

**B**

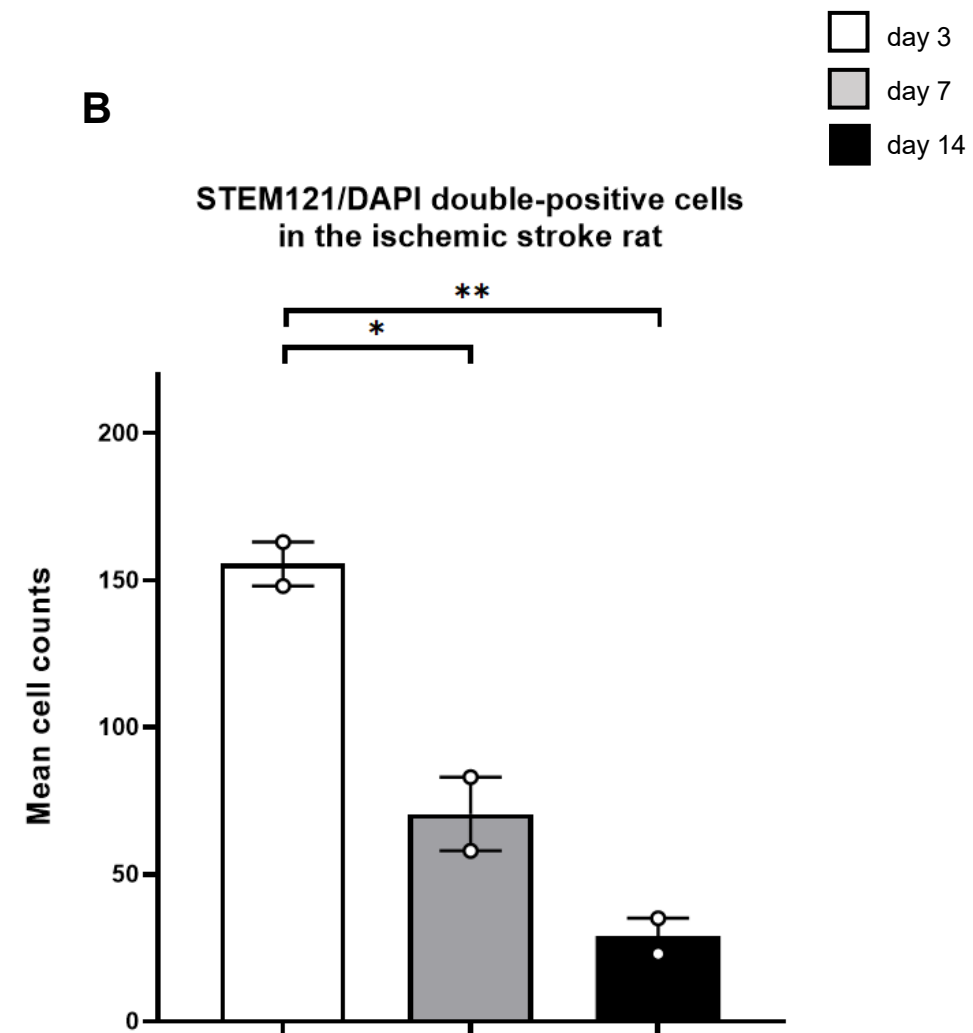

SB623+Ex group

A

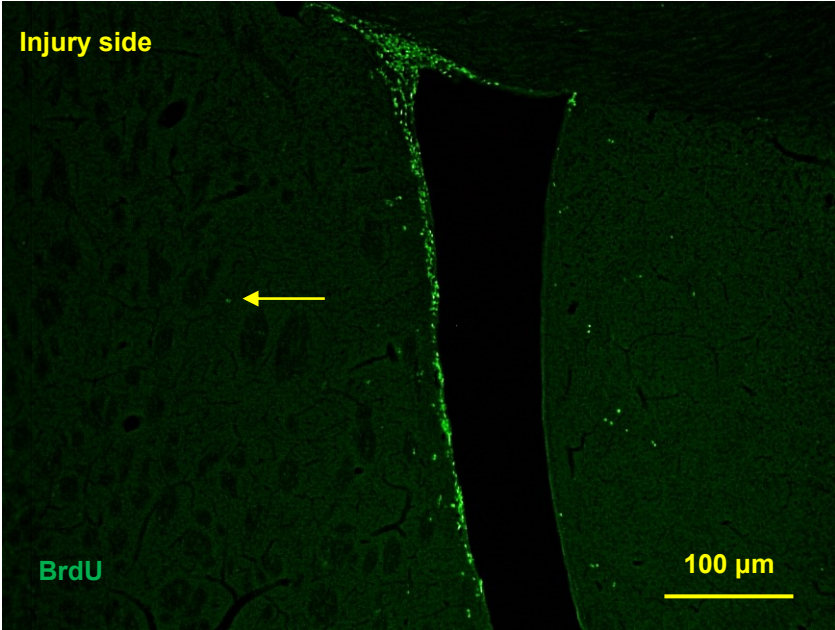

B

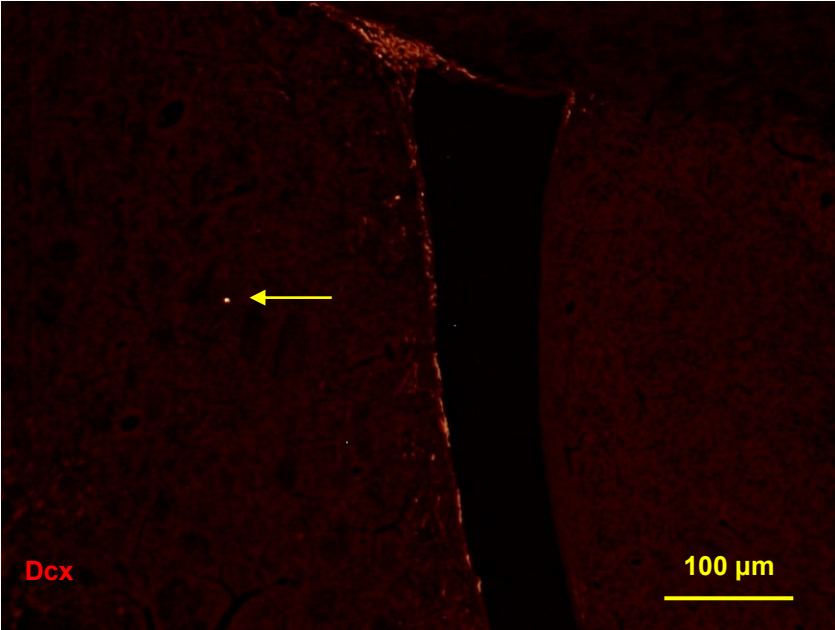

C

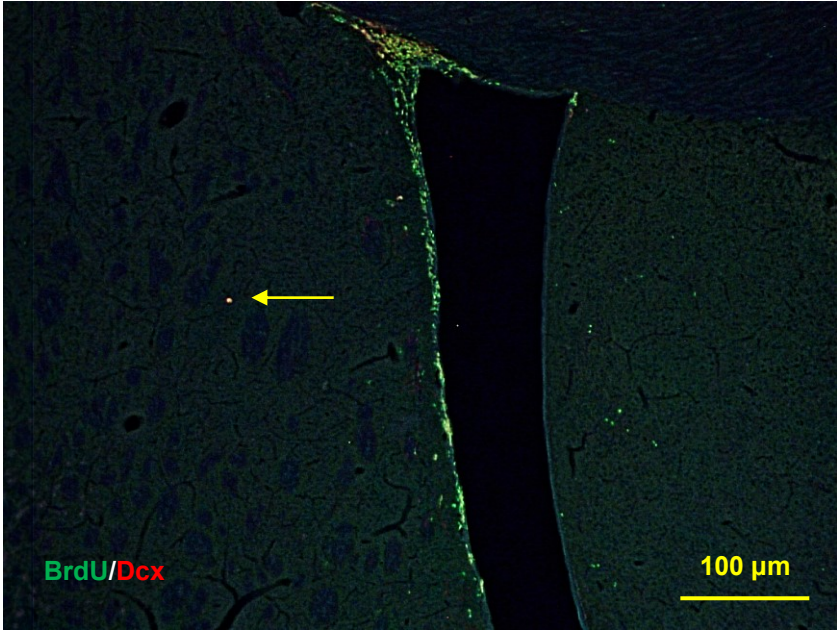

Supplement: Supplementary file 1 — Additional file 1. Figure S1: Illustration of counting BrdU/Dcx double-positive cells in the SZV and DG. (a, b) Brdu/Dcx double-positive cells were counted in 16 areas (4 areas × 2 sections × 2 hemispheres) for the SVZ, and in 32 areas (8 areas × 2 sections × 2 hemispheres) for the DG in each rat. Abbreviation: SVZ: subventricular zone; DG: dentate gyrus; BrdU: 5-bromo2’-deoxyuridine, Dcx: Doublecortin. Figure S2: Immunofluorescent staining for angiogenesis in the ischemic boundary zone. Representative lower-magnification photographs of laminin-positive area in SB623+Ex group are shown. We measured the area of laminin-positive structures of randomly captured images (500 × 500 μm square) in the ischemic cortex from two different IBZ slices (0 and 0.5 mm anterior to the bregma) (left: low magnification, right upper and right lower: high magnification). scale bar = 100 μm. Abbreviation: IBZ: ischemic boundary zone. Figure S3: Immunofluorescent staining for the viability of SB623 cells in the ischemic stroke rat after intracerebral transplantation. (a) Immunofluorescent staining for STEM121/DAPI double-positive cells shows SB623 cells at day 3 in the ischemic stroke rat after intracerebral transplantation (white arrow) (upper: low magnification, lower: high magnification). scale bar = 100 μm. (b) The number of STEM121-positive cells in the ischemic stroke rat at day 3 after transplantation tended to be higher than that at day 7(each group: n = 2) (F (2, 5) = 50.2, p < 0.01) (mean ± SE, *p < 0.05, **p < 0.01 vs. day 3). Abbreviation: DAPI: 4’ 6-diamidino-2-phenylindole. Figure S4: Migration of transplanted SB623 cells in SB623+Ex group. Immunofluorescent staining for BrdU (A), Dcx (B), and BrdU/Dcx (C) in the SVZ shows SB623 cells migrated toward the injury side. Yellow arrows show the most migrated BrdU/Dcx double-positive cell. scale bar = 100 μm. Abbreviation: SVZ: subventricular zone; BrdU: 5-bromo2’-deoxyuridine, Dcx: Doublecortin. [file 13287_2023_3236_MOESM1_ESM.pdf]
